# Identifying human pre-mRNA cleavage and polyadenylation factors by genome-wide CRISPR screens using a dual fluorescence readthrough reporter

**DOI:** 10.1093/nar/gkae240

**Published:** 2024-04-08

**Authors:** Zuyao Ni, Nujhat Ahmed, Syed Nabeel-Shah, Xinghua Guo, Shuye Pu, Jingwen Song, Edyta Marcon, Giovanni L Burke, Amy Hin Yan Tong, Katherine Chan, Kevin C H Ha, Benjamin J Blencowe, Jason Moffat, Jack F Greenblatt

**Affiliations:** Donnelly Centre for Cellular and Biomolecular Research, University of Toronto, 160 College Street, Toronto, ON M5S 3E1, Canada; Donnelly Centre for Cellular and Biomolecular Research, University of Toronto, 160 College Street, Toronto, ON M5S 3E1, Canada; Department of Molecular Genetics, University of Toronto, 1 King's College Circle, Toronto, ON M5A 1A8, Canada; Donnelly Centre for Cellular and Biomolecular Research, University of Toronto, 160 College Street, Toronto, ON M5S 3E1, Canada; Department of Molecular Genetics, University of Toronto, 1 King's College Circle, Toronto, ON M5A 1A8, Canada; Donnelly Centre for Cellular and Biomolecular Research, University of Toronto, 160 College Street, Toronto, ON M5S 3E1, Canada; Donnelly Centre for Cellular and Biomolecular Research, University of Toronto, 160 College Street, Toronto, ON M5S 3E1, Canada; Donnelly Centre for Cellular and Biomolecular Research, University of Toronto, 160 College Street, Toronto, ON M5S 3E1, Canada; Donnelly Centre for Cellular and Biomolecular Research, University of Toronto, 160 College Street, Toronto, ON M5S 3E1, Canada; Donnelly Centre for Cellular and Biomolecular Research, University of Toronto, 160 College Street, Toronto, ON M5S 3E1, Canada; Department of Molecular Genetics, University of Toronto, 1 King's College Circle, Toronto, ON M5A 1A8, Canada; Donnelly Centre for Cellular and Biomolecular Research, University of Toronto, 160 College Street, Toronto, ON M5S 3E1, Canada; Department of Molecular Genetics, University of Toronto, 1 King's College Circle, Toronto, ON M5A 1A8, Canada; Program in Genetics and Genome Biology, The Hospital for Sick Children, Toronto, ON Canada; Donnelly Centre for Cellular and Biomolecular Research, University of Toronto, 160 College Street, Toronto, ON M5S 3E1, Canada; Department of Molecular Genetics, University of Toronto, 1 King's College Circle, Toronto, ON M5A 1A8, Canada; Program in Genetics and Genome Biology, The Hospital for Sick Children, Toronto, ON Canada; Donnelly Centre for Cellular and Biomolecular Research, University of Toronto, 160 College Street, Toronto, ON M5S 3E1, Canada; Department of Molecular Genetics, University of Toronto, 1 King's College Circle, Toronto, ON M5A 1A8, Canada; Donnelly Centre for Cellular and Biomolecular Research, University of Toronto, 160 College Street, Toronto, ON M5S 3E1, Canada; Department of Molecular Genetics, University of Toronto, 1 King's College Circle, Toronto, ON M5A 1A8, Canada; Donnelly Centre for Cellular and Biomolecular Research, University of Toronto, 160 College Street, Toronto, ON M5S 3E1, Canada; Department of Molecular Genetics, University of Toronto, 1 King's College Circle, Toronto, ON M5A 1A8, Canada; Program in Genetics and Genome Biology, The Hospital for Sick Children, Toronto, ON Canada; Institute for Biomaterials and Biomedical Engineering, University of Toronto, Toronto, ON Canada; Donnelly Centre for Cellular and Biomolecular Research, University of Toronto, 160 College Street, Toronto, ON M5S 3E1, Canada; Department of Molecular Genetics, University of Toronto, 1 King's College Circle, Toronto, ON M5A 1A8, Canada

## Abstract

Messenger RNA precursors (pre-mRNA) generally undergo 3′ end processing by cleavage and polyadenylation (CPA), which is specified by a polyadenylation site (PAS) and adjacent RNA sequences and regulated by a large variety of core and auxiliary CPA factors. To date, most of the human CPA factors have been discovered through biochemical and proteomic studies. However, genetic identification of the human CPA factors has been hampered by the lack of a reliable genome-wide screening method. We describe here a dual fluorescence readthrough reporter system with a PAS inserted between two fluorescent reporters. This system enables measurement of the efficiency of 3′ end processing in living cells. Using this system in combination with a human genome-wide CRISPR/Cas9 library, we conducted a screen for CPA factors. The screens identified most components of the known core CPA complexes and other known CPA factors. The screens also identified CCNK/CDK12 as a potential core CPA factor, and RPRD1B as a CPA factor that binds RNA and regulates the release of RNA polymerase II at the 3′ ends of genes. Thus, this dual fluorescence reporter coupled with CRISPR/Cas9 screens reliably identifies *bona fide* CPA factors and provides a platform for investigating the requirements for CPA in various contexts.

## Graphical Abstract

**Graphical Abstract:**
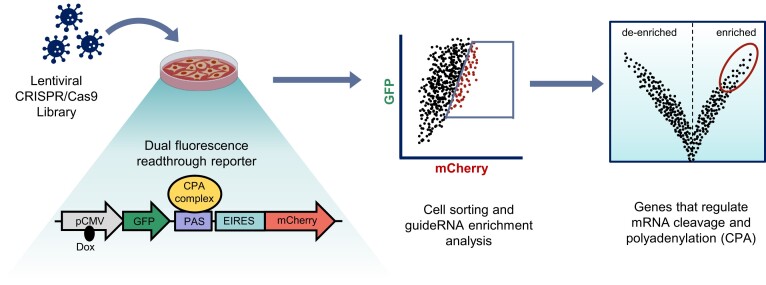

## Introduction

Eukaryotic messenger RNA precursors (pre-mRNAs) generally undergo 3′end processing by cleavage and polyadenylation (CPA). CPA is essential for mRNA stability, cytoplasmic export, and efficient translation (1–6). This 3′ end processing can also regulate gene expression through alternative polyadenylation (APA) (2,7,8), and defects in 3′ end processing can lead to various diseases (9–11). CPA consists of endonucleolytic cleavage at a polyadenylation site (PAS), followed by the synthesis of a polyadenosine (poly(A)) tail (12–14). It requires *cis*-regulatory elements present in the pre-mRNA (15,16), including a sequence related to the AAUAAA hexamer, and is regulated by the *trans*-acting CPA machinery and associated proteins (16). The canonical core CPA machinery consists of the four multi-protein complexes CPSF (cleavage and polyadenylation specificity factor), CSTF (cleavage stimulation factor), CFI (cleavage factor I) and CFII (cleavage factor II) (16–18). Important CPA factors also include the CPA scaffolding protein SYMPK (19), the RNA polymerase II (RNAP II) carboxy-terminal domain (CTD) (20–23), and PAP ((Poly(A)-polymerase)) (24–26), as well as PABP ((Poly(A) Binding Proteins) (27).

An increasing number of other human CPA factors have been identified in various contexts and biological processes (16). For example, RBBP6 was discovered as a new component of the CSTF complex (28), while components of other complexes, including CDK12 in a RNAP II CTD kinase complex (29), PPP1R10 in the PNUTS-PP1 (protein phosphatase 1 nuclear targeting subunit-protein phosphatase 1) complex (30,31), and the VIRMA subunit of the RNA N^6^-methyladenosine (m6A) methyltransferase complex (32), also known as the m^6^A-METTL Associated Complex (MACOM) (33,34), have been found to play an important role in 3′ end processing. Notably, proteomic studies have identified over 80 proteins associating with RNA containing the canonical PAS motif AAUAAA (17). These discoveries indicate that the factors involved in CPA are more numerous than previously thought and highlight the importance of identifying new CPA factor in various contexts.

To date, most of the CPA factors have been discovered through biochemical and proteomic studies. Transcription readthrough reporter systems have also proven effective in the investigation of mRNA 3′ end processing (35–37). However, single readthrough reporter systems are unable to distinguish promoter effects from those caused by a PAS, since the only reporter is located downstream of both the promoter and the PAS (36,38). This problem can be overcome by using a dual reporter system, in which one reporter is located between the promoter and PAS to serve as an internal control for assessing expression variation caused by effects on the promoter, while the second reporter is located downstream of the PAS to monitor readthrough effects resulting from the PAS (39). The expression ratio for the two reporters can then provide an accurate measure of the efficiency of 3′ end processing events between the reporters.

CRISPR (clustered regularly interspersed palindromic repeats)-based genetic screens are powerful tools for identifying genes that are important for various biological processes, including proliferation (40,41), tumorigenesis (40,42), drug resistance (43), and cancer metastasis (44). However, such screens have not been applied to identify the sequences or factors involved in 3′ end processing. Here, we describe combining the use of a genome-wide, pooled CRISPR/Cas9 knockout library with a dual fluorescence readthrough reporter system to genetically identify human CPA factors. Our screens effectively identified most of the well-characterized factors and some new ones. Our study demonstrated that a functional genome-wide CRISPR/Cas9 screen coupled with a dual fluorescence readthrough reporter system can be an efficient, accurate and reliable method for the identification of CPA factors.

## Materials and methods

### Cell culture

HEK293 cells (Flp-In 293 T-REx cell line) were obtained from Life Technologies (Invitrogen, catalogue number R780-07). Cell cultures were maintained in Dulbecco's modified Eagle's medium (DMEM) (Wisent Inc., catalogue number 319-005-CL) supplemented with 10% FBS (fetal bovine serum) (Wisent Inc., catalogue number 098150), sodium pyruvate, non-essential amino acids, and 100 IU/ml of penicillin and 100 μg/ml of streptomycin (Wisent Inc., catalogue number 450-201-EL, or Gibco catalogue number 15140-122). Cell counts were done using the Invitrogen Countess Automated Cell Counter hemocytometer (Invitrogen AMQAX1000) in a Countess cell counting chamber slide (Invitrogen by Thermo Fisher Scientific, catalogue number 100078809) for cells stained with 4% Trypan blue (Invitrogen by Thermo Fisher Scientific, catalogue number T10282).

### Antibodies

The following antibodies were used in this study: GFP, Abcam rabbit polyclonal, catalogue number 290, or Invitrogen rabbit monoclonal, catalogue number G10362; IgG negative controls, Millipore rabbit polyclonal, catalogue number 12-370, or Invitrogen catalogue number 10500C; RPRD1B (157–170), Sigma-Aldrich rabbit polyclonal, catalogue number SAB1102247; POLR2A (N-20), Santa Cruz polyclonal, catalogue number sc-899; CDK12, Proteintech rabbit polyclonal, catalogue number 26816-1-AP; CCNK, Bethyl Laboratories rabbit polyclonal, catalogue number A301-939-T; GAPDH, Invitrogen mouse monoclonal, catalogue number 39-8600. All antibodies were used at a dilution of 1:1000 to 1:5000 in 5% BSA (bovine serum albumin, BioShop, catalogue number ALB005) for western blotting, and 5 μg was used in chromatin immunoprecipitation (ChIP) experiments. All the commercial antibodies have been validated for the relevant species and applications, as shown on the manufacturers’ websites.

### Western blots

Cells were suspended in SDS protein sample buffer ((140 mM Tris (EMD, catalogue number 9230) pH 6.8, 4% SDS (BioShop, catalogue number SDS001), 20% glycerol (BioShop, catalogue number Gly002.4), 0.02% bromophenol blue (BioShop, catalogue number BRO222.5), 1:100 diluted 2-mercaptoethanol (Bio Basic Canada, catalogue number MB0338) and incubated on ice for 15 min. Lysates were boiled for 5 min and clarified in a microfuge at 15 000 g for 2 min at 4ºC. The supernatant was run on a 10% SDS polyacrylamide gel and transferred to an activated PVDF membranes (Bio-Rad, catalogue number 162–0177) in a Tris-glycine transfer buffer containing 20% methanol (Caledon Laboratory Chemicals, catalogue number 6701-7-40) in a Gel Transfer Cell (BioRad catalogue number 1703930). Horseradish peroxidase-conjugated goat anti-mouse IgG (H + L) (Thermo Fisher Scientific, catalogue number 31430) or anti-rabbit IgG (H + L) (Thermo Fisher Scientific, catalogue number 31460) secondary antibodies were used at a dilution of 1:5000. Blots were developed using the ECL Western Blotting Detection Reagent (Cytiva Amersham, catalogue number RPN2106) and visualized by using MicroChemi 4.2 (Bio-Imaging Systems).

### Construction of a dual fluorescence readthrough reporter

We carried out the following cloning steps to construct a GFP-mCherry dual fluorescence readthrough reporter (Supplementary Figure S1). First, a 540 bp IRES fragment from plasmid pIGCN21 (45) (from the laboratory of Dr N. Copeland) was PCR amplified using the forward primer IRES.E.F1 (5′-ATAGAATTCACGTTACTGGCCGAAGCCGCT-3′, EcoRI site underlined) and reverse primer IRES.B.R1 (5′- ATAGGATCCTTTTTCAAAGGAAAACCACGT-3′, BamHI site underlined), digested with EcoRI (NEB, catalogue number R0101S) and BamHI (NEB, catalogue number R0136S) and ligated to the EcoRI/BamHI-digested plasmid pAcGFP1-N3 (Clontech Laboratories Inc., catalogue number 632484), to generate a pIRES-GFP1 vector. Second, a 760 bp fragment containing mCherry (714 bp)-NLS (nuclear localization signal, 21 bp) was PCR amplified from the pLentiGuide mCherry-NLS-P2A-puro plasmid (46) (obtained from Dr. Daniel Durocher, The Lunenfeld-Tanenbaum Research Institute, Mount Sinai Hospital, Toronto, Canada) using the forward primer mCherry.NLS.BamHI.F: (GATAGGATCCGCCACCATGGTGAGCAAGGGCGAG, BamHI site underlined) and reverse primer NLS.mCherry.NotI.R: (ATATGCGGCCGCTCA*CACTTTCCGCTTTTTCTTGGG* CTTGTACAGCTCGTCCATGCC, NotI site underlined, NLS is underlined and italic), digested by using BamHI and NotI (NEB, catalogue number R0189S), and ligated to the BamHI/NotI-digested pIRES-AcGFP1 vector to generate a pIRES-mCherry/NLS vector. The NLS fused to mCherry in this construct allows the nuclear localization of mCherry. Similarly, a 767 bp fragment of BamHI (10 bp)-GFP (720 bp)/NLS (21 bp)-NotI (15 bp) was PCR amplified from pAcGFP1-N3 plasmid using the forward primer NLS.G.BamHI.F (ATAGGGATCCATCATGGTGAGCAA BamHI site underlined) and reverse primer NLS.G.NotI.R: (GTACTCTAGATCA*CACTTTCCGCTTTTTCTTGGG*CTTGTACAGCTCATCCATGCCGT, NotI site underlined, NLS is underlined and italic), digested by using BamHI and NotI, and ligated to the BamHI/NotI-digested pIRES-AcGFP1 vector to generate a pIRES-GFP/NLS vector. This step added an NLS downstream of GFP. Third, a 1696 bp PCR fragment containing AflII-IRES (540 bp)-mCherry (1577 bp)-NLS (21 bp)-BclI was PCR amplified from pIRES-mCherry/NLS vector by using forward primer For-AflII (GAGCTTAAGAGCTGGTTTAGTGAACCGTCA, AflI site underlined) and reverse primer BclI-Dist (ACCTGATCAGGACAAACCACAACTAGAATGC, BclI site underlined), digested by using AflII (NEB, catalogue number R0520S), and BclI (NEB, catalogue number R0160S), and ligated to AflII/BclI-digested doxycycline inducible Flp-In T-REx vector (pDEST-pCDNA5/FRT/TO-eGFP, Addene, ID 52506) to generate a pIRES-mCherry/NLS-F vector. In this vector, the CMV promoter is activated by doxycycline, and a Flp element is responsible for site-specific genome integration (47). Fourth, a 759 bp fragment containing an AflII -AcGFP (741 bp)-NLS (21 bp)-NheI fragment was PCR amplified from the pIRES-GFP/NLS vector using the forward primer GFP.AflII.F1 (ATACTTAAGATGGTGAGCAAGGGCGCCGAGCT, AflII site underlined) and reverse primer GFP.NheI.R1 (ATAGCTAGCTCACACTTTCCGCTTTTTCTTG, NheI site underlined), digested by using AflII and NheI, and ligated to the AflII/NheI digested pIRES-mCherry/NLS-F vector to generate the pGFP/NLS-IRES-mCherry/NLS-F vector. In this vector, GFP serves as an internal control. In addition, an NLS was added downstream of mCherry. Fifth, since the sequence of the IRES (540 bp) is shorter than that of EIRES (595 bp) (Supplementary Figure S2A), and the IRES-mediated translation effect is relatively weak (Supplementary Figure S2B), the IRES was replaced by EIRES. A 603 bp fragment containing EcoRI-EIRES (595 bp)-BamHI was PCR amplified from the MSCV-GPS-GAW plasmid (48) (obtained from Dr Stephen J. Elledge, Harvard University) using the forward primer IRES.EMCV.Eco.F1 (ATAGAATTCGCCCCTCTCCCTCCCCCCCCCCTAA, EcoRI site underlined) and reverse primer IRES.EMCV.Bam.R1 (ATAGGATCCTGTGGCCATATTATCATCGTG, BamHI site underlined), digested by using EcoRI and BamHI, and ligated to the EcoRI/BamHI digested pGFP/NLS-IRES-mCherry/NLS-F vector to generate the pGFP/NLS-EIRES-mCherry/NLS-F (pGECF) vector. Indeed, the expression of EIRES-initiated mCherry is stronger than that mediated by IRES (Supplementary Figure S2B). Finally, PAS DNA elements defined as described previously (49) were PCR amplified from human genomic DNA by using NheI and EcoRI recognition sequence-containing forward and reverse primers, respectively, digested with these two restrict enzymes and ligated into the pGECF vector by T4 DNA ligase (New England BioLabs Inc., catalogue number M0202S). All the above clones were verified by DNA sequencing.

### Cloning and purification of recombinant proteins

Full-length RPRD1B (S2-D326), as well as its CTD-interacting domain (CID) (S2-P135) and its coiled-coil domain (171–304), were cloned into the pET28GST-LIC vector (GenBank accession EF456739) with the In-Fusion Dry-down Mix (Clontech, catalogue number S3533) according to the manufacturer's instructions. The identities of all plasmid constructs were verified by sequence analysis. pET28GST-LIC cloned plasmids were transformed into BL21 Star One Shot *Escherichia coli* (Thermo Fisher Scientific, catalogue number C601003), and protein expression was induced by the addition of 1 mM Isopropyl β-d-1-thiogalactopyranoside (IPTG) (Sigma-Aldrich, catalogue number I5502) to 1 l of culture medium when the bacteria had reached an optical density (OD_600_) of 0.4. Proteins were expressed for 12 h at 14°C with shaking, and then the bacteria were pelleted (approximately 10–15 g) and frozen at −80°C. The pellet was then thawed on ice, and the bacteria were lysed in 20–70 ml of suspension buffer (50 mM HEPES, pH 7.5, 500 mM NaCl, 5 mM imidazole, 5% glycerol, 1 mM protease inhibitor, 1 mM DTT and 6.25 units/ml of benzonase) by sonication with the setting of 10 s on and 10 s off, totally or 10 min. Undissolved debris was pelleted at 15000 rpm for 1 hour at 4°C. Extracts containing the 6 × His-tagged recombinant proteins were incubated with 4–14 ml (1:5) Ni-NTA Superflow resin (Qiagen, catalogue number 30450), depending on the protein expression levels. The beads were then washed four times with wash buffer (50 mM HEPES, pH 7.5, 500 mM NaCl, 30 mM imidazole and 5% glycerol), followed by elution with 5–20 ml of elution buffer (50 mM HEPES pH 7.5, 500 mM NaCl, 250 mM imidazole and 5% glycerol). Eluted proteins were further purified by Superdex 75 or 200 gel filtration utilizing the AKTA purifier (GE Healthcare Bio-Science). All proteins were concentrated by using Amicon Ultra-4 Centrifugal Filter Devices (Millipore) to 10–50 mg/ml in a buffer containing 10 mM HEPES pH 7.5, and 150 mM NaCl and stored at –80°C.

### GFP-tagged proteins

A Gateway-compatible RPRD1B (OHS1770-9385393) entry clone was obtained from the human ORFeome library (Open Biosystems) and sequence verified. The entry clone was cloned into a Flp-In T-REx GFP vector (pDEST pcDNA5/FRT/TO-eGFP, Invitrogen, catalogue number K6010) by LR reaction as described previously (50,51). In brief, the LR reaction was conducted using 75 ng of RPRD1B entry clone DNA, 75 ng GFP-Tag destination vector DNA, 6 μl Tris EDTA (TE) buffer, and 2 μl Gateway™ LR clonase II enzyme mix (Invitrogen, catalogue number 11791) in a total volume of 20 μl at 25°C for 1–2 h, followed by the addition of 1 μl protease K (Sigma-Aldrich, catalogue number P2308) at 37°C for 10 min. LR reaction products were transformed into 50 μl Subcloning Efficiency DH5α cells (Invitrogen, catalogue number 18265) followed by incubation in LB buffer at 37°C overnight. Plasmid DNA was purified from selected colonies by using the Presto Mini Plasmid Kit (Geneaid, catalogues number PDH300). GFP-tagged RPRD1B DNA were transfected into Flp-In T-REx HEK293 cells by using FuGENE (Roche, catalogue number11 814 443 001) according to the manufacture's instructions, and stably expressing cell lines were selected by the addition of 2 μg/ml hygromycin B (Invitrogen, catalogue number 10687-010).

### Stable RPRD1B knockout cell lines

CRISPR/Cas9 targeting plasmids pCMV-Cas9-2A-GFP (Sigma-Aldrich, catalogue number CAS9GFPP) expressing a Scrambled guide RNA (CGCGATAGCGCGAATATATTNGG) or RPRD1B (GGGACCCATCGTCTCCGTGTGG) guide RNA (gRNA) were purchased from Sigma-Aldrich. 2 μg of plasmid DNA was transfected into HEK293 cells using FuGene reagent according to the manufacturer's instructions. Twenty-four hours after transfection, cells were sorted by BD FACS Melody sorter (Temerty Faculty of Medicine Flow Cytometry Facility, University of Toronto), and single GFP-positive cells were plated into 48-well plates. The expression level of RPRD1B in each expanded clone was detected by western blotting.

### Lentiviral CRISPR/Cas9-mediated knockouts

CRISPR/Cas9 plasmid LentiCRISPRv2 (kindly provided by Dr Jason Moffat at the University of Toronto; Addgene plasmid catalogue number 52961) was constructed to express the gRNAs of AAVS1 #1 GGGGCCACTAGGGACAGGAT; AAVS1 #2 GTCACCAATCCTGTCCCTAG); CPSF1 #1 CATGGAGAACTCCAGACCGG; CPSF1 #2 CAAGCTCGAGCTTCTCCCGG; CPSF2 #1 CAACTTGGAGAAGATAGCAA; CPSF2 #2 ACTTTCCGACAGCATACGGG; CPSF3 #1 AATAGGAGATCAATCTCAGC; CPSF3 #2 GATCTCAATCATGAACATGG; CPSF4 #1 GTTTGACTTGGAGATCGCGG; CPSF4 #2 GAGTTTCTTACCTTTGCCGC; WDR33 #1 TGAAAGTGAGTCCATTCCAC; WDR33 #2 ATGTTGACAGCAGACCACGG; FIP1L1 #1 CTAGTGTCGGAGCTGAGCGG; FIP1L1 #2 TGTGCACATGCACGTCCCAT; RPRD1B #1 TGGGTCCCGCGTGCTTGCGG; RPRD1B #2 CAAGAACGAAGTGTGTATGG; CCNK#1 TGAACCGAGCGCCCTCTCGG; CCNK#3 GATGGTTCGGAGCTTTGAGA; CDK12 #2 CTTCACAGAAACCTGTACAG; or CDK12 #4 TCATGTTAACAACACTTCGG, according to the Toronto KnockOut version 3.0 (TKOv3) gRNA library (52). Each cloned gRNA was transfected into HEK293T cells to produce lentiviral particles, which were subsequently used to infect HEK293 cells. One day after infection, cells were subjected to puromycin selection (final concentration 2 μg/ml; Sigma catalogue number p8833) for 3 days and grown for additional 3 days before being harvested. The knockout efficiency of each gRNA was examined by western blot.

### Pooled CRISPR/Cas9 library lentivirus production and titre determination

The pooled CRISPR gRNA Library lentiviruses were prepared as described previously (53). In brief, approximately 8 × 10^6^ HEK293T cells were seeded per 15-cm plate in DMEM containing high glucose, pyruvate and 10% FBS. Twenty-four hours after seeding, the cells were transfected with 8 μg lentiviral lentiCRISPRv2 vector DNA containing the TKOv3 gRNA library (Addgene, catalogue number 90294), 4.8 μg packaging vector psPAX2 (Addgene, catalogue number 12260), 3.2 μg envelope vector pMD2.G (Addgene, catalogue number 12259), 48 μl X-tremeGene 9 DNA Transfection Reagent (Roche, catalogue number 066365787001) and 1.4 ml Opti-MEM medium (Gibco, catalogue number 31985-070). Twenty-four hours after transfection, the medium was replaced with serum‐free, 1.1 g/100 ml BSA DMEM that contains 1% penicillin and 1 μg/ml streptomycin. Virus-containing medium was collected 48 h after transfection, centrifuged at 1500 rpm for 5 min, aliquoted and frozen at −80°C.

For the determination of viral titres, 3 × 10^6^ HEK293 cells seeded in 15-cm plates were transduced with different dilutions of the pooled CRISPR/Cas9 library lentiviruses along with 4 μg/ml of polybrene (Hexadimethrine bromide, Sigma catalogue number H9268), in a total of 20 ml medium. After 24 h, the virus-containing medium was replaced with 20 ml of fresh DMEM medium containing 2 μg/ml of puromycin, and cells were incubated for an additional 48 h. Multiplicity of infection (MOI) of the titrated virus was determined 72 h post‐infection by comparing percentage survival of puromycin-selected cells with these infected but not selected with puromycin (puro-minus controls).

### Pooled CRISPR/Cas9 library lentiviral infection

Approximately a total of 10^8^ Flp-In T-REx HEK293 cells grown in five 15-cm plates (20 × 10^6^ cells per 15-cm plate) were infected with the pooled CRISPR/Cas9 library lentiviruses at a MOI of 0.3 along with 4 μg/ml of polybrene. Twenty-four hours later, infected cells were selected with 2 μg/ml of puromycin for 72 h to achieve a 200-fold coverage of the TKOv3 library. Cells were then induced with 1 μg/ml of doxycycline (Sigma-Aldrich, catalogue number D9891) for 4 days for the expression of both GFP and mCherry.

### Fluorescence-activated cell sorting (FACS) and cell analysis

After the pooled CRISPR/Cas9 library lentiviral infection and doxycycline induction, cells were washed once with 1× PBS (Wisent Inc., catalogue number 211-010-LL), harvested by treatment with 0.05% trypsin (Wisent Inc., catalogue number 325-542-CL), resuspended in Sorting Buffer (1 × PBS, 0.2% BSA and 0.2 μg/ml DAPI (Sigma catalogue number 10236276001), 1 mM EDTA (BioShop, catalogue number EDT111.500)) and passed through a 40-μm mesh (Fisherbrand, catalogue number 22363545). Cells were sorted by using the Aria IIu Sorter (Department of Immunology, University of Toronto). Approximately 0.5 × 10^6^ cells per sample with an increased mCherry/GFP ratio were collected into 2 ml of 50% FBS containing DMEM spiked with 5 × 10^6^ wild-type HEK293 cells. Twenty million unsorted cells were harvested on the same day. The FACS experiments were performed in biological quadruplicates.

For cell analysis, 1 × 10^6^ of dual fluorescence readthrough reporter-integrated HEK293 cells were grown in 6-well plates, infected with gRNA-containing lentiviruses, induced by doxycycline, washed and harvested as in FACS. Harvested cells were passed through a tube with a Cell-Strainer Cap (Falcon, catalogue number 352235). Single cells were analyzed using the BD FACS Aria IIu or BD FACS Melody cell sorter (Temerty Faculty of Medicine Flow Cytometry Facility, University of Toronto).

### Preparation of sequencing libraries and Illumina sequencing

Genomic DNA was purified from a total of 20 × 10^6^ unsorted cells and approximately 0.5 × 10^6^ sorted cells spiked with 5 × 10^6^ wild type cells using the Wizard Genomic DNA Purification Kit (Promega, catalogue number A1125), and quantified by Qubit 2.0 Fluorometer (Invitrogen by Life Technologies) using the reagents of Qubit dsDNA BR Assay Kit (Invitrogen, catalogue number Q32850). Sequencing libraries were prepared from 50 μg (approximately 100-fold coverage of the TKOv3 library) of the extracted genomic DNA in two PCR steps. For the first PCR, a total of 14 PCR reactions were carried out using 2 × NEBNext Ultra II Q5 Master Mix (NEB, catalogue number M0544L) to amplify the gRNA inserts, with each 50 μl reaction containing 3.5 μg (a total of 50 μg) of genomic DNA. Forward primer v2.1.F1 GAGGGCCTATTTCCCATGATTC, and reverse primer v2.1.R1 GTTGCGAAAAAGAACGTTCACGG were used for the first PCR. The PCR cycles included 98°C for 30 s, followed by 25 (for unsorted control samples) to 38 (for sorted samples) cycles of 98°C for 10 s, 66°C for 30 s and 72°C for 15 s, and an extension at 72°C for 2 min. PCR products of 599 bp were visualized by running 5 μl in a 1% agarose (Invitrogen by Life Technologies, catalogue number 16500-500) gel, and all 14 reactions for each genomic DNA sample were pooled together.

For the second PCR, the gRNA was amplified using 5 μl of the above pooled first PCR products as a template in a 50 μl reaction with NEBNext Ultra II Q5 Master Mix by using the primers harboring Illumina TruSeq adapters i5 (with staggered sequences) and i7 barcodes (Supplementary Figure S3A, B). The PCR cycles included 98°C for 30 s, followed by 10 cycles of 98°C for 10 s, 55°C for 30 s and 65°C for 15 s, and an extension at 65°C for 5 min. PCR products of ∼203 bp (Supplementary Figure S3C) were visualized by running all 50 μl in a 1.8% agarose gel, and the bands were excised. These barcoded library DNAs were purified from the agarose gel slices using the GenepHiow Gel/PCR kit (Geneaid, catalogue number DFH300) and quantified by Qubit 2.0 Fluorometer using the reagent of QuantiFluor ONE dsDNA system (Promega, catalogue number E4871).

The resulting libraries were sequenced on an Illumina HiSeq2500 (Donnelly Sequencing Centre, University of Toronto, Canada) to a depth of 10–20 million, using single-read sequencing, and completed with standard primers for dual indexing with HiSeq SBS Kit v4 reagents. The first 21 cycles of sequencing were dark cycles, or base additions without imaging. The actual 36-base read began after the dark cycles and contained 2 index reads, in which i7 was read first, followed by the i5 sequences.

### Microscopy analysis

The expression of GFP and mCherry in HEK293 cells was visualized by using an Olympus CKX41 microscope.

### RNA extraction and quantitative RT-qPCR

Total RNA was extracted using Trizol Reagent (Invitrogen, catalogue number 15596018) as per the manufacturer's instructions and cDNA was synthesized using 2.5 μg of total RNA with the SuperScript VILO Kit (Invitrogen, catalogue number 11754). qPCR was performed with PowerUp SYBR Green Master Mix (Appliedbiosystems, catalogue number A25742) on a ViiA7 by Life Technologies Real Time PCR System (Applied Biosystems). The qPCR program included 40 cycles of 95°C for 15 s and 55°C for 30 s, and a final cycle (95°C for 15 s and then 60°C). The following primers were used for RT-qPCR: GFP forward CACATGAAGCAGCACGACTT and reverse GATGCGATTCACCAGGGTAT; mCherry forward TCAGTTCATGTACGGCTCCA and reverse CCGTCCTCGAAGTTCATCAC; MYB exon 4 forward CCTCCTGGACAGAAGAGGAA and reverse CAGGCAGTAGCTTTGCGATT; MYB upstream of PAS forward CGCTGGTCATGTGAGACATT and reverse CTTGGTGCTGCTCTCAACTG; MYB downstream of PAS forward AGGTGGTGTCTTGCCATCTT and reverse TCACACCTGTAATCCCAGCA; YKT6 exon 4 forward TGAATTCTCCAAGCAAGTCG and reverse ATCTACTGAGGTGACCATCCA; YKT6 upstream of PAS forward CTGAGAGCACCCACTGTCCT and reverse AGGTAAACCAGCCAGGAGGT; YKT6 downstream of PAS forward ATCCTGGAAGGCAGAGACCT and reverse GCACCCTCTGAACAAAGCTC; CPEB2 exon 2 forward AAACAGTCTCCCTGGAGCAA and reverse TCCCATGTTTCCGGTTCTAC; CPEB2 upstream of PAS forward TTGCTGCCCAAAAGTATGAC and reverse AGCCAGATGCAACAGGGATA; CPEB2 downstream of PAS forward CCAGGAAACATGAAGACATGG and reverse TGTGTGAAGCTTTTTAGCCACA; GEMIN7 exon 1 forward TCGGTGAGTACAAGGTGGTG and reverse GTCACTCAGGGCGTTGCAC; GEMIN7 upstream of PAS forward CAATGCAAACTCCAGTGAACA and reverse CAGGAACCTCTGGCCTCA; GEMIN7 downstream of PAS forward GGGAGCTGAATATTCGTTATTTG and reverse CGGAACTGCAGACTATGAGG; RUNX1 exon 3 forward GGCTGGCAATGATGAAAACT and reverse CCGACAAACCTGAGGTCATT; RUNX1 upstream of PAS forward CACGCGCTACCACACCTAC and reverse GAGGCGCCGTAGTACAGGT; and RUNX1 downstream of PAS forward TGCTTACAAAATGGCTGCCT and reverse CCCAGTGCCCATCATTCAAC.

### iCLIP-seq procedure

iCLIP-seq was performed as described previously (54), with modifications introduced in iCLIP-1.5 (55). Briefly, HEK293 cells expressing GFP-tagged RPRD1B or CPSF1 were grown in 15 cm culture plates. Cells were UV crosslinked using 0.15 J/cm^2^ at 254 nm in a Stratalinker 1800 (Stratagene) prior to harvesting. One ml of the lysates, prepared in iCLIP lysis buffer (50 mM Tris–HCl, pH7.4; 100 mM NaCl; 1% Igepal CA-630; 0.1% SDS; 0.5% sodium deoxycholate) supplemented with protease/phosphatase inhibitors (Protease Inhibitor Cocktail Set III; Calbiochem/Merck, catalog number: 539134-1SET), was incubated with Turbo DNase (Life Technologies, catalogue number AM2238) and RNase I (1:250; Ambion, catalogue number AM2294) for exactly 5 min at 37°C with shaking at 1400 rpm in a thermomixer to obtain RNA fragments of an optimal size range and digest genomic DNA. The lysates were then immunoprecipitated using 6 μg anti-GFP antibody conjugated with Protein G dynabeads (Life Technologies, catalog number: 10004D). Beads were washed with iCLIP high salt buffer (50 mM Tris–HCl, pH7.4; 1000 mM NaCl; 1 mM EDTA; 1% Igepal CA-630; 0.1% SDS; 0.5% sodium deoxycholate), and RNA dephosphorylation was performed using FastAP (Life Technologies, catalogue number EF0652) and T4 polynucleotide kinase (New England Biolabs, catalogue number M0202L). Pre-adenylated L3 adaptors were ligated to the 3′-ends of RNAs using the enhanced CLIP ligation method (56), as detailed in iCLIP-1.5 (55). The immunoprecipitated RNA was 5′-end-labeled with ^32^P using T4 polynucleotide kinase. Protein–RNA complexes were separated using 4–12% BisTris–PAGE and transferred to a nitrocellulose membrane protran BA85 (VWR, catalogue number 732-4174). The membrane corresponding to RNA fragments of optimal range was excised and proteins were digested with proteinase K (Thermo Fisher, catalogue number 25530049). RNA was reverse transcribed into cDNA using barcoded iCLIP primers. The cDNA was size selected (low: 70–85 nt, middle: 85–110 nt, and high: 110–180 nt), and circularized using CircLigase™ II ssDNA Ligase (Lucigen, catalogue number CL4115K) with betaine at a final concentration of 1 M. The reaction mixture was incubated for 2 h at 60°C. Circularized cDNA was digested at the internal BamHI site for linearization, and PCR amplified using AccuPrime SuperMix I (Thermo Fisher, catalog number 12344040). iCLIP libraries were gel purified using the GenepHlow Gel//PCR kit, and mixed at a ratio of 1:5:5 from the low, middle, and high fractions prior to sequencing on an Illumina HiSeq2500 platform (Donnelly Sequencing Centre, University of Toronto, Canada) to generate single-end 51 nucleotide reads with 40 million read depth per sample. The following barcoded primers were used for iCLIP-seq:

- Replicate 1 RtCLIP13: /5Phos/NNTCCGNNNAGATCGGAAGAGCGTCGTGgatcCTGAACCGC
- Replicate 2 RtCLIP16: /5Phos/NNTTAANNNAGATCGGAAGAGCGTCGTGgatcCTGAACCGC

RPRD1B iCLIP-seq was performed as part of our large-scale study to investigate the RNA binding maps of numerous RBPs (RNA binding proteins).

### Electrophoretic mobility shift assays (EMSA)

Biotin-labeled RNA probes were incubated in 20 μl binding buffer (40 mM Tris pH8.0, 30 mM KCl, 1 mM MgCl_2_, 0.01% NP-40, 1 mM DTT, 100 ng/ml yeast tRNA), and 0–1 μg purified recombinant proteins at 30°C for 30 min to 1 h, as previously described (57,58). The reactions were then cooled on ice and heparin (final concentration of 200 μg/ml) was added. The reaction products were resolved on a 4–6% native polyacrylamide gel in 0.5 × TBE (90 mM Tris pH 8.3, 90 mM borate, 2 mM EDTA) running buffer at 100 V in a cold room and transferred to a positively charged nylon membrane. The membrane was then blotted with Streptavidin-HRP and visualized by MicroChemi4.2. The following biotin-labeled probe sequences were used:

- GEMIN7 (Inside 3′ UTR, chr19:45594389-45594489+): AUUUGAUUUCAUUUUGGGGCGGGCGGUGGCUCGUGCUGGGUCACGUGGUCGUGCCAAGCGCUCCUCCUGUUGCCCCACCUGUGGUUGCUGUGGACUGCAC .
- GEMINI7 (Protein Coding Exon 2, chr19:45583169-45583280+)

GCGGCUUCUCUGUUGACAACUCAGCUGGUUCCACACCCUGGCAAUUGUGAAGAGUUGGCCAAAUGUUUGUCCACUGAGCUGAUCUCCUCUCUGGAGCACC

### Chromatin immunoprecipitation and sequencing (ChIP-seq)

ChIP was performed as previously described (59). Briefly, approximately 20 × 10^6^ of stable RPRD1B or Scrambled knockout HEK293 cell line were crosslinked with 1% formaldehyde (Sigma, catalogue number F8775), harvested in lysis buffer (50 mM Tris–Cl pH 8.1, 10 mM EDTA, 1% SDS, 1 mM phenylmethylsulfonyl fluoride (PMSF, Sigma, catalogue number *P*-7626), 10 μg/ ml aprotinin (Sigma, catalogue number A-1153), and 10 μg/ml leupeptin (Sigma, catalogue number L-2884)) and sonicated by using a Branson Sonifier 450 (USA, Danbury, CT) with the setting of time: hold, dutycycle: 30%, output control: 3, 25 pulse each time for 7 times. Subsequently, RNAP II was immunoprecipitated from the lysates with 5 μg of RNAP II (N-20) antibody followed by crosslink reversal at 65°C overnight and DNA precipitation by using the QIAEX II Gel Extraction Kit (Qiagene, catalogue number 20051). Libraries were sequenced on an Illumina HiSeq 2500 (Donnelly Sequencing Centre, University of Toronto, Canada) to a depth of 20 million 51-nucleotide single end reads.

### gRNA enrichment analysis

Sequence reads from all samples were preprocessed by trimming staggers from the 5′ends and anchors from the 3′ends using an in-house perl script. The length of resulting sequences was mostly 20 bp, and the minimal length was 19 bp. Sequence reads that did not contain staggers and/or anchors were discarded. The preprocessed sequences were then aligned to the TKOv3 gRNA library (containing 70 948 gRNAs targeting 18053 protein coding genes (4 gRNAs/gene)) using Bowtie v1.1.2 (parameters -v2 -m1 -p4 –sam-nohead). The number of reads that were aligned to each gRNA in TKOv3 in each sample was counted using an in-house perl script. The R package DESeq2 was used to determine genes that show significantly different read counts between sorted and unsorted populations grown in parallel (60). Genes that had less than 4 guides were excluded from the analysis (608 genes). In total, 17 445 genes were considered for further analysis. Each guide for a gene was treated independently. To account for differences in sequencing depth, the read counts for each guide were scaled by a factor equal to the sum of the reads for a guide across all genes divided by the geometric mean of the sums of all guides. Statistical *P*-values were adjusted using the Benjamini and Hochberg method for multi-testing correction (61).

### iCLIP-seq data analysis

iCLIP-seq data analysis was performed as reported in our previous publications (62,63). In brief, 51-nt iCLIP-seq raw reads, which consist of 3 random positions, a 4-nt multiplexing barcode, and another two random positions, followed by the cDNA sequence, were first de-duplicated based on the first 45 nt. Then the random positions, barcodes, and any 3′-bases matching Illumina adaptors were removed. Reads shorter than 25 nt were discarded, and the remaining reads were trimmed to 35 nt. These steps were performed using Trimmomatic (64). Reads were mapped to the human genome/transcriptome (Ensembl annotation hg19) using TopHat (65) with default settings. Reads with a mapping quality <3 were removed from further analysis.

Crosslink induced truncation sites (CITS) from individual iCLIP replicates for RPRD1B and CPSF1 were called using the CLIP Tool Kit (CTK) (66) with default settings. CPSF1 iCLIP-seq data was acquired from our previous publication (62) (GEO accession code: GSE 165772). CITS with FDR ≤0.01 were considered significant and were merged from the two replicates for further analysis. Metagene plots along the transcript and peak distribution across genomic regions (analyzing 5′ and 3′UTRs separately) were generated using the R package GenomicPlot (URL: https://github.com/shuye2009/GenomicPlot).

### ChIP-seq data analysis

ChIP-seq data analysis was performed essentially as described previously (59). Briefly, Illumina adaptor sequences were removed from the 3′ ends of 51-nt reads, and the remaining reads were mapped to the human genome hg19 using Bowtie 2 with default settings. After removal of duplicate reads, peaks were called jointly on immunoprecipitated and input samples with MACS2 (version 2.1.2) (67,68). ChIP-seq signal intensity (log2(ratio-over-input)) in transcription termination site (TTS) regions (from 1000 bp upstream to 3000 bp downstream of the TTS) of 11340 actively expressed genes (FPKM > 1) in HEK293 cells was analyzed using ‘GenomicPlot’. Wilcox test was used to determine the statistical significance of differences in RNAP II signal intensity between perturbed cells (CRISPR/Cas9-mediated RPRD1B knockout or RPRD1B overexpression) and their corresponding control cells (Scramble knockout or GFP overexpression, respectively).

### Statistical analysis

Statistical analyses were performed for RT-qPCR data using one tailed Student's *t* test. Data are given as means ± SD by two or three independent experiments.

## Results

### A dual fluorescence readthrough reporter to measure the effects of 3′ end processing

**Figure 1.**
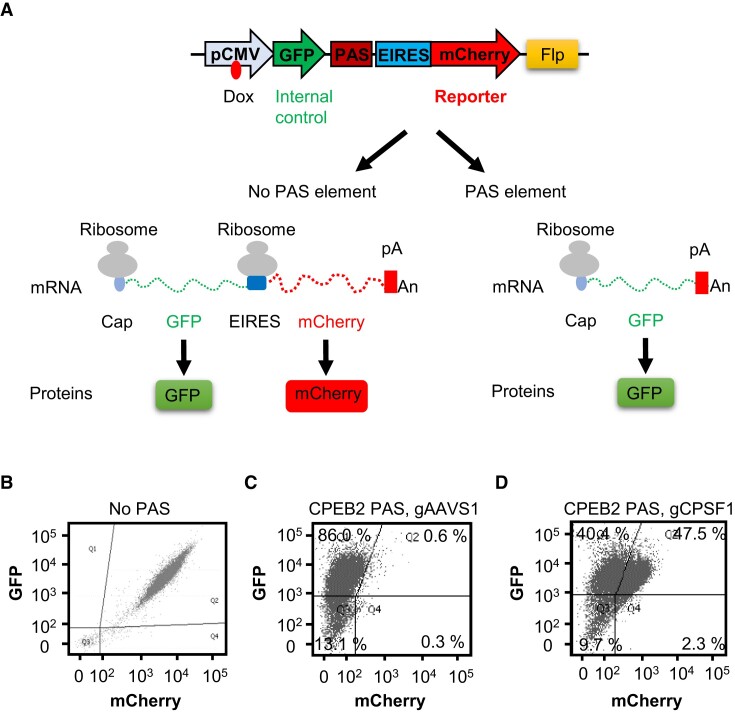
A dual fluorescence readthrough reporter to measure the effects of 3′ end processing. (**A**) Schematic illustration of the dual fluorescence readthrough reporter. In the construct without a PAS, a doxycycline-inducible CMV promoter drives transcription through both fluorescent reporters, GFP and mCherry. Ribosomes dock at the 5′ end cap of the bicistronic mRNA to translate GFP or at the EIRES to translate the uncapped mCherry portion of the transcript. In the construct with a PAS, the transcription is terminated upstream of mCherry, whereas the GFP portion of the transcript remains intact. The expression ratio between the mCherry and GFP reporters measures transcriptional readthrough effects that reflect 3′ end processing. Dox: doxycycline. (**B**) Constant mCherry/GFP ratio in the dual fluorescence readthrough reporter without a PAS. (**C**) A distal PAS of the CPEB2 gene decreases the mCherry/GFP ratio in the dual fluorescence readthrough reporter. (**D**) CRISPR/Cas9-mediated depletion of CPSF1 increases the mCherry/GFP ratio in the dual fluorescence readthrough reporter containing the distal PAS of the CPEB2 gene. Expression in (B)–(D) was induced by the addition of 2 μg/ml doxycycline for 4 days in HEK293 cells, and each dot represents the expression in one living cell. GFP and mCherry expressions in 10 000 cells were analyzed by FACS in each experiment.

To efficiently identify CPA factors, we established a tandem readthrough reporter system (Figure 1A and Supplementary Figure S1A, B). Since the luciferase reporters used previously in a tandem reporter system (39) could not be used to sort single cells via FACS, we replaced them with fluorescent reporters (Figure 1A and Supplementary Figure S1A, B). In our dual fluorescent reporter system, *Aequorea coerulescens* GFP was cloned between the promoter and a PAS to serve as an internal control, while mCherry was cloned downstream of the PAS to monitor readthrough effects (Figure 1A and Supplementary Figure S1A, B). This cassette contains an encephalomyocarditis-virus internal ribosome entry site (EIRES) upstream of mCherry (69,70), permitting the translation of two fluorescent proteins from one mRNA transcript (Figure 1A, Supplementary Figures S1A, B, and S2A, B). Because CPA leads to subsequent downstream termination by RNAP II (71), CPA would prevent expression of the downstream reporter, mCherry. This cassette was further cloned into the Flp-In T-REx (Flp recombinase integration and tetracycline-regulated mammalian expression) vector that allows for Flp-mediated integration into the genome at a specific site of Flp-In T-REx HEK293 cells (Figure 1A and Supplementary Figure S1A) (47,72,73). The Flp-In T-REx vector also contains a doxycycline inducible cytomegalovirus (CMV) promoter (Figure 1A and Supplementary Figure S1A, B). After induction with doxycycline, the expression of the two fluorescent reporters in single living cells could then be assessed by FACS.

As expected, in the absence of a PAS, the expression levels of both GFP and mCherry, as measured by FACS, varied substantially as a consequence of varying levels of promoter activity in different cells (Figure 1B). In contrast, the mCherry/GFP ratio in different cells remained relatively constant (Figure 1B), demonstrating its independence from promoter activity. Insertion of a 432 bp DNA fragment containing the distal PAS of the CPEB2 gene (Supplementary Figure S4A) between the GFP and EIRES-mCherry cassettes substantially reduced the expression of mCherry, but not that of GFP, resulting in a decreased mCherry/GFP ratio in 86.0% of the cells (Figure 1C). The mCherry/GFP ratio from the readthrough reporters after insertion of an irrelevant DNA element did not change (data not shown). Similarly, insertion of a 339 bp DNA fragment containing the distal PAS of the CCND2 gene substantially decreased the mCherry/GFP ratio in 94.7% of the cells (Supplementary Figure S4B, C). These results indicated that the decreased mCherry/GFP ratio in a PAS-containing dual fluorescence readthrough reporter can reproduce the expected effects of 3′ end processing.

Next, we assessed whether the dual fluorescence readthrough reporter could detect the effects of depleting an essential CPA factor. The cells were infected with lentiviruses that transduced Cas9 and a guide RNA (gRNA) designed to knock out the CPSF1 gene. Since CPA factors are generally essential for cell survival (74), preliminary experiments (not shown) were done to establish the time needed after lentivirus infection to adequately deplete CPSF1 without causing cell death. After induction of the reporter genes with doxycycline, FACS then showed that CRISPR/Cas9-mediated CPSF1 knockout led to a substantial increase of the mCherry/GFP ratio in 47.5% and 41.3% of the cells containing the PAS of the CPEB2 and CCND2 genes, respectively (Figure 1D, and Supplementary Figure S4C). Similar results were obtained after infecting the cells with lentiviruses that transduced gRNAs for the other CPSF subunits (i.e CPSF2, CPSF3, CPSF4, WDR33 and FIP1L1) (Supplementary Figure S4D). In contrast, the mCherry/GFP ratio in cells infected with lentiviruses that transduced an AAVS1 control gRNA remained at background levels of 0.6% and 0.2% in the two PAS containing cell lines, respectively (Figure 1C and Supplementary Figure S4C). These results demonstrated that the dual fluorescence readthrough reporter system responds effectively to the loss of a CPA factor and, therefore, that a change in the mCherry/GFP ratio in the system can be a reliable indicator of 3′ end processing in mammalian cells.

### Genome-wide CRISPR screens to identify CPA factors

**Figure 2.**
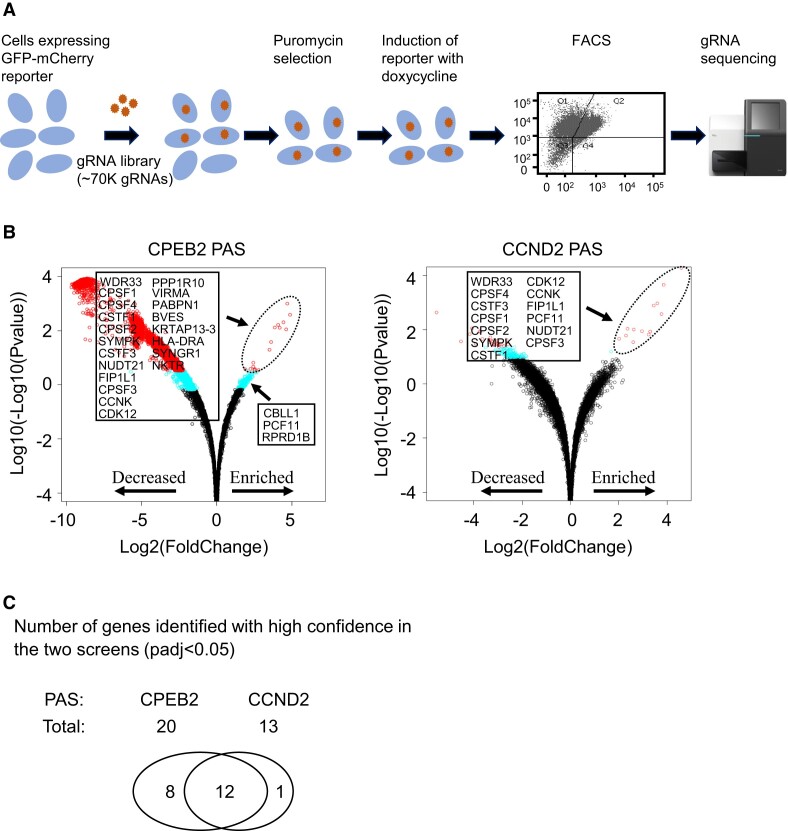
CRISPR/Cas9 screens identified CPA factors. (**A**) CRISPR/Cas9 screening pipeline. Flp-In T-REx HEK293 cells expressing the GFP-mCherry readthrough reporter with an inserted PAS were infected with the pooled lentiviral human TKOv3 gRNA library followed by puromycin selection. After doxycycline-induced expression of the two reporters, cells were sorted by FACS. Genomic DNA was isolated from unsorted cells and sorted cells that exhibited an increased mCherry/GFP ratio, and the gRNAs were amplified and subjected to sequencing. (**B**) CRISPR/Cas9 screen results for HEK293 cell lines expressing the GFP and mCherry reporters separated by the distal PAS from the CPEB2 (left) and CCND2 (right) genes. Scatterplots (volcano plots) display pairwise comparisons of the gRNAs between sorted cells that exhibited an increased mCherry/GFP ratio and unsorted cells. Red circles: *P*_adj_ < 0.05. Blue circles: *P*_adj_ < 0.25, but >0.05. Black circles: *P*_adj_ > 0.25. Experiments were performed in biological quadruplicate. *P*_adj_ values were determined using the Benjamini–Hochberg adjustment for multiple comparisons. (**C**) Genes identified in both screens with high confidence. The gRNAs of 12 genes were enriched with high confidence (*P*_adj_ < 0.05) in sorted cells with increased mCherry/GFP ratio as compared with unsorted cells in both screens.

Next, we performed genome-wide pooled CRISPR/Cas9 screens using our dual fluorescence readthrough reporter system (Figure 2A). To identify the commonly used CPA factors, we investigated the responses to the distal PAS of two different genes, CPEB2 and CCND2 (Supplementary Figure S4A, B). We selected these genes because their APA regulation is important for oncogenic activation (75). For the screens, we employed the TKOv3 CRISPR/Cas9 library, which contains 70948 gRNAs targeting 18053 protein-coding genes (52), nearly always with 4 guides each. The TKOv3 library also included 142 control guides targeting EGFP, LacZ, and Luciferase, for a total of 71 090 guides (40,52).

After lentiviral infection of the cells with the TKOv3 library for 7 days and doxycycline induction of the dual fluorescence system, FACS was conducted to isolate cells with an increased mCherry/GFP ratio, followed by isolation of genomic DNA (Figure 2A). The DNA fragments containing gRNA sequences were amplified by polymerase chain reaction (PCR) and then subjected to Next Generation Sequencing (Figure 2A). The enrichment of gRNAs in sorted cells with increased mCherry/GFP ratio compared to unsorted cells was then analysed statistically using DESeq2 (60) and corrected for multiple hypothesis testing (61). The enrichment of genes with gRNAs that met a threshold of *P*_adj_ < 0.05 (adjusted *P* value in multiple comparisons) was considered to have a very high level of confidence, with few, if any, false positives, while genes with *P*_adj_ <0.25 but >0.05 were considered to have relatively lower levels of confidence and to contain a significant number of false positives. These genes with *P*_adj_ <0.25 gRNAs were considered as potential CPA factors, and those enriched in both screens seemed likely to serve as common CPA factors. The genes most strongly enriched (*P*_adj_ < 0.05) in both screens seemed likely to encode common core CPA factors. Genes that were strongly enriched (*P*_adj_ < 0.05) in only one of the two screens may represent gene-specific CPA factors, whereas those weakly enriched (*P*_adj_ < 0.25, but >0.05) in only one of the two PAS screens may represent gene-specific, weak or partially redundant CPA factors, or else false positive hits.

The screens identified a total of 174 and 14 genes based on a cut-off of *P*_adj_ <0.25 for the PAS of CPEB2 and CCND2, respectively (Figure 2B, and Supplementary Tables S1 and S2). Of these, 13 genes were enriched in both screens (*P*_adj_ < 0.25) (Figure 2B, C, Table 1 and Supplementary Tables S1 and S2), suggesting that they are likely common CPA factors. Twenty and 13 genes were enriched with high confidence (*P*_adj_ < 0.05) in the PAS of CPEB2 and CCND2, respectively (Figure 2C, Table 1 and Supplementary Tables S1 and S2), suggesting that they are likely to be strong CPA factors. Twelve of these highly enriched genes were found in both screens (Figure 2C, Table 1 and Supplementary Tables S1 and S2), indicating that they are likely to be common and core CPA factors. As expected, these common and core hits contained 10 canonical core CPA factors, including all six of the CPSF subunits (CPSF1, CPSF2, CPSF3, CPSF4, WDR33 and FIP1L1), as predicted by the preliminary FACS results with the dual fluorescent reporters (Figure 1D and Supplementary Figure S4D), two (CSTF1 and CSTF3) of the four CSTF subunits, one (NUDT21) of the three CFI subunits, and the CPA scaffold protein SYMPK (Figure 2C, Table 1 and Supplementary Tables S1 and S2) (76). WDR33 was the most highly enriched gene in both screens (Figure 2B, Table 1 and Supplementary Tables S1 and S2), consistent with its direct interaction with the canonical PAS hexamer AAUAAA (77,78). One well-characterized CPA factor, the PCF11 subunit of CFII, was identified with high confidence in the CCND2 PAS screen (*P*_adj_ < 0.05) but relatively low confidence in the CPEB2 PAS screen (*P*_adj_ < 0.25, but *P*_adj_ > 0.05) (Figure 2B, Table 1, and Supplementary Tables S1 and S2), possibly because different amounts of PCF11 may be needed for the two PAS (79). Put together, the screens identified most of the well-characterized core CPA factors with high confidence. Notably, the above results also indicated that the screens with the different PAS had different sensitivities. In the following descriptions and investigations, we will focus more on the hits identified with the more sensitive CPEB2 PAS.

**Table 1.** Common CPA factors identified in the screens for the PAS of CPEB2 and CCND2 genes

			CPEB2 PAS	CCND2 PAS	
#	Complexes	Genes	*P*_adj_	*P*_adj_	PAS binding*
1	CPSF	WDR33**	6.24E-162	1.22E-69	+
2		CPSF1	1.09E-102	2.50E-15	+
3		CPSF2	1.37E-12	1.76E-10	+
4		CPSF3	3.23E-07	0.0145424	+
5		CPSF4	8.38E-100	4.61E-36	+
6		FIP1L1	5.92E-08	0.0007598	+
7	CSTF	CSTF1	2.27E-19	5.93E-05	+
8		CSTF3	1.02E-08	1.04E-16	+
9	CFI	NUDT21	4.87E-08	0.0042814	+
10	CPA scaffold	SYMPK	1.52E-09	3.57E-05	+
11	CFII	PCF11	0.06263***	0.0010556	+
12	CTD kinase	CCNK	7.11E-05	0.0001607	−
13		CDK12	0.000815	0.0001589	−

*Affinity purification and mass spectrometry results from Shi *et al.* (17).

**The highest confidence enrichment in the two screens.

***P_adj_ <0.25, but >0.05.

+: Binding to RNA containing a canonical PAS with the AAUAAA hexamer. −: No PAS binding was observed.

The Poly(A) Binding Protein PABPN1, a known CPA factor (27), was identified only with the more sensitive CPEB2 PAS with high confidence (*P*_adj_ < 0.05) (Figure 2B, C, and Supplementary Table S1). Similarly, PPP1R10 (also known as PNUTS), the scaffold of a PNUTS–PP1 complex, was also identified only with the same PAS with high confidence (*P*_adj_ < 0.05) (Figure 2B, C, and Supplementary Table S1). This result is in agreement with previous observations that PPP1R10/PNUTS plays an important role in dephosphorylating SUPT5H to slow elongation by RNAP II, thereby facilitating transcription termination (31).

The gRNAs for VIRMA (Vir Like M6A Methyltransferase Associated) and CBLL1 (Cbl proto-oncogene like 1, or HAKAI), two subunits of the MACOM complex that creates m6A in RNA (33,34), were enriched in the CPEB2 PAS screen with high (*P*_adj_ < 0.05) and low (*P*_adj_ < 0.25, but >0.05) confidence, respectively (Figure 2B, and Supplementary Table S1). These results are in line with previous studies demonstrating that VIRMA associates with CFI (i.e. NUDT21/CPSF5 and CPSF6) in the regulation of APA (32), and CBLL1 interacts with the CPSF1, CPSF2, and FIP1L1 subunits of CPSF and SYMPK (33,80), as well as with PSF (PTB-associated splicing factor) (81,82), which recruits the exonuclease XRN2 (83) important for transcription termination (84). Furthermore, the WTAP subunit of MACOM has previously been shown to interact with CPSF (80). Thus, results obtained from previous proteomic studies and the genetic screens in this study suggest that CBLL1, like WTAP and VIRMA, may be a *bona fide* CPA factor, perhaps because m6A modifications in 3′ UTRs is important for CPA (32).

Neither the CSTF2 and CSTF2T subunits of CSTF, nor the CPSF6 and CPSF7 subunits of CFI, was found in either screen, likely due to their redundant effects (85–87). Similarly, PAP was not identified, possibly because of compensatory effects of the four poly(A) polymerases (i.e. PAP, Neo-PAP, Star-PAP and TPAP) (88). The reason for the absence of CLP1, a CFII component, is currently unknown and warrants further investigation. However, false negative results in CRISPR/Cas9 screens of the kind we carried out can also simply reflect either ineffective gRNAs for particular genes or absence of gRNAs for certain genes in the library.

Overall, as described here, the use of a dual fluorescence readthrough reporter in conjunction with pooled CRISPR/Cas9 library screens proved to be an effective method for identifying most of the non-redundant core CPA factors.

### The CCNK/CDK12 complex as a functional requirement for CPA

**Figure 3.**
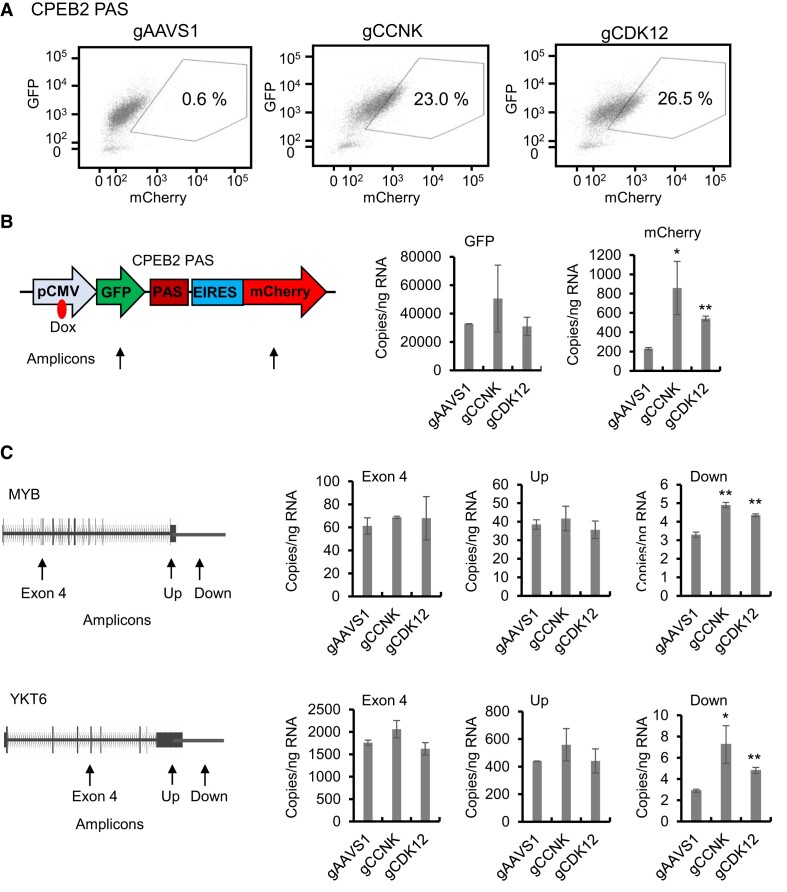
CCNK and CDK12 in 3′ end processing. (**A**) CRISPR/Cas9-mediated depletion of CCNK or CDK12 increases the mCherry/GFP ratio in HEK293 cells expressing the dual fluorescence readthrough reporter containing the distal PAS of the CPEB2 gene. 10000 cells were analyzed by FACS. (**B**) Depletion of CCNK or CDK12 specifically increases the expression of mCherry RNA relative to that of GFP RNA in HEK293 cells expressing the dual fluorescence readthrough reporter containing the distal PAS of the CPEB2 gene. RNA expression levels were measured by RT-qPCR using primers targeting the indicated reporter regions in cells infected with lentiviruses transducing the indicated gRNAs. (**C**) Depletion of CCNK or CDK12 increases RNA expression downstream of the PAS in endogenous genes. RNA expression from the MYB and YKT6 genes was measured by RT-qPCR using primers targeting the indicated gene regions in cells infected with lentiviruses transducing the indicated gRNAs. Up: upstream of PAS. Down: downstream of PAS. The above results represent two biological replicates. *P< 0.05, **P< 0.01 as compared to the gAAVS1 control.

The gRNAs of both CDK12 (cyclin dependent kinase 12) and CCNK (cyclin K), like these of the core CPA factors, were found to be significantly enriched with high confidence (*P*_adj_ < 0.05) in both screens (Figure 2B, C, Table 1 and Supplementary Tables S1 and S2), suggesting that both likely play an important role in efficient 3′ end processing. These results are consistent with previous demonstrations that CDK12 is important for 3′ end processing (89). Although CCNK, like CDK12, was known to also have the opposite effect by suppressing premature intronic polyadenylation (29,90,91), its role as a requirement for 3′ end processing has remained elusive. To verify these screen results, particularly those for CCNK, we first examined the mCherry/GFP ratio in CCNK- or CDK12-depleted cells (Supplementary Figure S5A). FACS showed that CRISPR/Cas9-mediated depletion of either CCNK or CDK12 increased the mCherry/GFP ratio in 23.0% and 26.5%, respectively, of the cells expressing the readthrough reporter with the distal CPEB2 PAS (Figure 3A). Similarly, knockout of CCNK or CDK12 increased the mCherry/GFP ratio in 8.1% and 3.8%, respectively, of the cells when the distal PAS of CCND2 was used (Supplementary Figure S5B). Again, these results indicated that the PAS of CPEB2 is more sensitive than that of CCND2 in the readthrough reporter system. To confirm these effects at the RNA level, we used RT-qPCR to measure RNA transcripts for the two reporters in CCNK- or CDK12-depleted cells with the CPEB2 PAS and found, as expected, that depletion of either CCNK or CDK12 significantly increased the RNA expression of mCherry relative to that of GFP, as compared to a gAAVS1 control (Figure 3B). To assess whether this effect also occurs for endogenous genes, we measured RNA expression for various regions of the MYB and YKT6 genes. Indeed, as compared to agAAVS1 control, depletion of either CCNK or CDK12 significantly increased RNA expression in the region downstream of the PAS relative to an internal exon or the region upstream of the PAS for the two genes that we tested (Figure 3C). Put together, these results demonstrated that CCNK, like CDK12, is important for efficient CPA at distal cleavage sites.

### Importance of RPRD1B for the regulation of 3′ end processing

**Figure 4.**
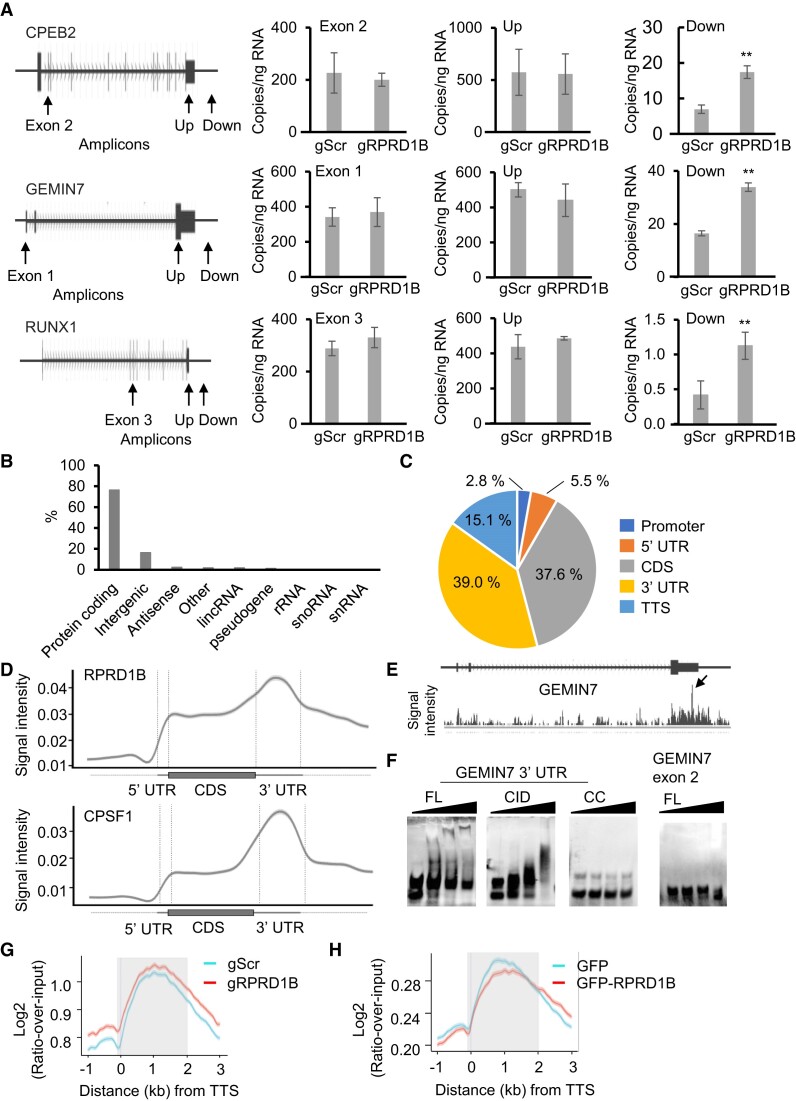
Involvement of RPRD1B in 3′ end processing. (**A**) Depletion of RPRD1B increases RNA expression downstream of the PAS of endogenous genes. RNA expression levels were measured by RT-qPCR using primers targeting the indicated gene regions after CRISPR/Cas9-mediated stable depletion using gRNAs targeting Scrambled (gScr) or RPRD1B (gRPRD1B). The results represent three biological replicates. **P< 0.01 as compared to Scrambled control. Up: upstream of PAS. Down: downstream of PAS. (**B**) RPRD1B iCLIP-seq peak annotation. Annotation of peaks to all type of genes is shown. Total number of peaks is 465247. (**C**) RPRD1B iCLIP-seq peak distribution. A pie chart was generated using the iCLIP-seq peaks at a threshold of FDR < 0.05. CDS: coding sequences; UTR: untranscribed regions; TTS: transcript termination sites. Absolute counts are shown. (**D**) RPRD1B binds preferentially to RNA at the 3′ends of genes. Standardized metaplot profiles showed the normalized PureCLIP peak densities of GFP-RPRD1B and GFP-CPSF1 along mRNA transcripts. Results from two biological replicates are shown. (**E**) RPRD1B binding profile on RNA from the GEMIN7 gene. Arrow: the highest peak of the RNA-binding region at the GEMIN7 gene. (**F**) RPRD1B directly binds to an RNA 3′end. Biotin-labeled RNA probes spanning the RPRD1B crosslinking sites in the 3′UTR or the irrelevant exon 2 region of the GEMIN7 gene were incubated with increasing concentrations (0–1 μg) of recombinant RPRD1B in EMSA assays. FL: full length RPRD1B. CID: CTD-interaction domain of RPRD1B. CC: coiled-coil domain of RPRD1B. (**G**) Knockout of RPRD1B increases RNAP II occupancy at the 3′ends of genes. Metagene profiles generated from RNAP II ChIP-seq results using N-20 antibodies in HEK293 cells after CRISPR/Cas9-mediated stable knockouts with gRNAs targeting Scrambled (gScr) or RPRD1B (gRPRD1B) are shown. (**H**) Over-expression of RPRD1B reduces the RNAP II occupancy at the 3′ ends of genes. Metagene profiles generated from ChIP-seq experiments performed as in (G) in HEK293 cells over-expressing either GFP-RPRD1B or GFP as a control are shown. In (G) and (H), density per million of ChIP fragments from 11 340 genes from two biological replicates is shown. TTS: transcript termination sites. Shadow: 3′UTR.

The gRNAs for RPRD1B (regulation of nuclear pre-mRNA domain containing 1B) were enriched in the CPEB2 PAS screen with low confidence (*P*_adj_ < 0.25, but >0.05) (Figure 2B, and Supplementary Table S1), suggesting that RPRD1B might serve as a weak factor important for CPA. To confirm this effect for endogenous genes, we measured RNA transcripts for various regions of the CPEB2, GEMIN7 and RUNX1 genes. Indeed, in comparison to a Scrambled control gRNA, stable knockout of RPRD1B significantly increased RNA expression in the regions downstream of the PAS relative to the regions upstream of the PAS or internal exons for the three genes that we tested (Figure 4A). Of note, the RT-qPCR results detected for the endogenous CPEB2 locus are consistent with the CRISPR screen results using the reporter system containing the CPEB2 PAS (Figure 2B, and Supplementary Table S1). These results are also in line with previous experiments indicating that RPRD1B associates with the transcription termination factor XRN2 (92), which is important for CPA, and its adaptor SFPQ (PSF) (83,92,93). Notably, RPRD1B contains a CTD-interacting domain (CID), which binds to the S2-phosphorylated RNAP II CTD (94,95). Our findings here are reminiscent of the regulation of 3′ end processing by several CID-containing proteins, including yeast Rtt103 (96) and *C. elegans* CIDS1/CIDS2 (97), as well as PCF11 (20).

The CID structure of RPRD1B is highly similar to that of PCF11 (94,98). Given that the CID of PCF11 interacts with the 3′ end RNA in the RNAP II transcription complex (99,100), we postulated that RPRD1B might also interact with RNA via its CID. To address this issue, we utilized individual-nucleotide resolution UV cross-linking and immunoprecipitation followed by high throughput sequencing (iCLIP-seq) (54,55) to investigate the RNA-binding preferences of RPRD1B. Our results showed that the majority (76.4%) of the RNAs bound by RPRD1B were protein-coding transcripts (Figure 4B), suggesting that RPRD1B primarily targets RNAP II-transcribed genes. Moreover, 39.0% of the iCLIP-seq peaks were found in the 3′UTRs (Figure 4C) and, consistently, our metagene analysis showed an enrichment of RPRD1B towards the 3′ ends of transcripts (Figure 4D). Although the RNA-binding profile of RPRD1B was similar to that of the core CPA factor CPSF1, we also observed excess RPRD1B signal downstream of the annotated cleavage sites (Figure 4D). These results are consistent with a role for RPRD1B in transcription termination and in line with RPRD1B being a hit in our screen.

To further explore the binding of RPRD1B to the 3′ends of target transcripts, we used recombinant RPRD1B in electrophoretic mobility shift assays (EMSA). The distal cleavage site of a target gene, GEMIN7, carrying RPRD1B binding sites (Figure 4E) was transcribed *in vitro*, and these transcripts were incubated with recombinant RPRD1B for electrophoresis on a non-denaturing gel. In these experiments, full-length RPRD1B and the CID of RPRD1B, but not the coiled-coil domain of RPRD1B, bound the GEMIN7 3′ UTR probe (Figure 4F). In contrast, a GEMIN7 Exon 2 probe, which had no RPRD1B iCLIP-seq peaks, was not bound by full-length RPRD1B in EMSA (Figure 4F). These results are consistent with our RT-qPCR results showing increased transcripts downstream of the PAS relative to upstream regions at the GEMIN7 locus (Figure 4A). These results indicated that RPRD1B, via its CID, can directly bind to the 3′end of a target mRNA.

**Figure 5.**
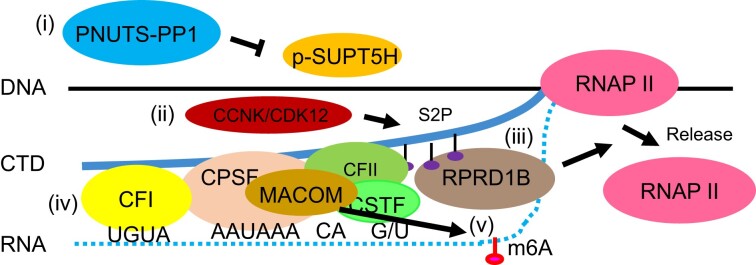
Identified CPA factors implicated in pre-mRNA 3′ end processing. Eukaryotic mRNA 3′ end processing is a multi-step process (126,127). In this study, we identified a variety of CPA factors implicated in multiple steps of 3′ end processing. (i) PPP1R10/PNUTS acts as a scaffold of a PP1 phosphatase complex (128), whose dephosphorylation of the elongation factor SUPT5H is important for 3′ end processing (31). (ii) CCNK/CDK12 kinase complex is responsible for S2-phophorylation of the RNAP II CTD (106,107), which exhibits its highest phosphorylation levels at the 3′ ends of genes (109). (iii) CID containing proteins PCF11 and RPRD1B link the S2-phophorylated RNAP II CTD (94,98) to the 3′ ends of mRNA (99,100), resulting in the dislodgement of RNAP II at the 3′ ends of genes. (iv) The canonical core CPA machinery comprising CPSF, CSTF, CFI, CFII and the scaffold protein SYMPK bind to the PAS and adjacent RNA sequences (4,17). (v) CBLL1-containing MACOM synthesizes the m6A that has been implicated in 3′ end processing (32,33).

Disruption of 3′ end processing usually leads to a readthrough effect, resulting in an accumulation of RNAP II downstream of the PAS (101–103). In line with this, RPRD1B was previously found to localize at the 3′ end of the CCND1 gene, and its loss led to increased occupancy by RNAP II downstream of the PAS of the CCND1 gene (104). To address whether this is a genome-wide effect, we performed ChIP-seq for RNAP II in HEK293 cells. ChIP-seq results showed that, in comparison with treatment with a Scrambled control gRNA, CRISPR/Cas9-mediated stable RPRD1B knockout led to an average genome-wide accumulation of RNAP II downstream of the transcript termination sites (TTS) of 11340 genes (Figure 4G). In contrast, and consistent with this result, over-expression of GFP-RPRD1B decreased the average RNAP II occupancy distal to the TTS genome-wide as compared to over-expression of a GFP control (Figure 4H). Thus, RPRD1B appears to have an average genome-wide effect in promoting the release of RNAP II at the 3′ ends of genes, in line with its genome-wide binding to the 3′ ends of mRNAs (Figure 4C, D). Therefore, our data supports a model (Figure 5) in which RPRD1B most likely serves as a bridge between RNAP II and the 3′ ends of pre-mRNA to control the release of RNAP II at the 3′ ends of genes. If RPRD1B can directly release RNAPII, such an activity would then be conserved between the CID-containing proteins RPRD1B and PCF11 (99,100).

## Discussion

### The importance of the CCNK/CDK12 kinase complex for CPA

Among all the well-documented CPA factors, four multi-protein complexes (i.e. CPSF, CSTF, CFI and CFII) primarily constitute the core CPA machinery. Most components of these complexes were identified with high confidence in our screens. These complexes contain the polypeptides that interact with the canonical PAS and/or adjacent RNA motifs (17). Like these core CPA factors, both CDK12 and CCNK were also enriched with high confidence. However, unlike the four well-characterized core CPA complexes, no interaction with the canonical PAS and/or adjacent RNA motifs has been found for either CCNK or CDK12 (17). Instead, the two proteins are tightly bound in a complex (105) and are responsible for S2 phosphorylation of the RNAP II CTD (106), which is crucial for 3′ end processing (21,107) and the interaction of certain CPA factors (e.g. PCF11) with the CTD (100).

Previous studies have already demonstrated that CDK12 is important for 3′ end processing (89) and can suppress premature intronic polyadenylation (29,90). These functions are thought to be primarily due to CDK12’s kinase activity responsible for CTD S2 phosphorylation (107,108), a hallmark of 3′ end processing (109). CCNK is a crucial partner and regulator of CDK12 (110), and the interaction of the two proteins is vital for the kinase activity responsible for CTD phosphorylation (106). Unlike CDK12, the importance of CCNK for 3′ end processing has not been investigated. However, in a recent study, CCNK, like CDK12, was found to suppress intronic polyadenylation in the androgen receptor gene (91). Despite these known activities, how CDK12 and CCNK can be important for CPA in 3′ UTRs but then suppress CPA in introns has not yet been addressed.

### RNA m6A in 3′ end processing

m^6^A is one of the most abundant modifications found in eukaryotic mRNAs (111), and it is primarily located near mRNA 3′ ends (112) and accompanies slowing by RNAPII (113). In addition to its involvement in various other biological processes (114), m6A plays an important role in 3′ end processing, particularly in APA (112). One of the underlying molecular mechanisms involves removal of blockages caused by RNA secondary structure (115). This enhances accessibility for RNA binding proteins that preferentially bind to single-stranded RNA motifs (116,117), a concept referred to as the ‘m6A structural switch’ (116).

MACOM is important for the synthesis of m^6^A and comprises multiple subunits, including CBLL1 (HAKAI), WTAP, VIRMA, ZC3H13 (KIAA0853), RBM15/15B and METTL3/14 (33). Previous proteomic studies have revealed that its CBLL1 and WTAP components associate physically with several subunits of CPSF (33,80), as well as with PSF (81,82), which recruits exonuclease XRN2 (83). Moreover, the VIRMA component has been shown to associate with CFI subunits NUDT21 (CPSF5) and CPSF6, thereby regulating APA (32). The screens in this study provided functional evidence for a potential role of CBLL1 in the regulation of 3′ end processing, further highlighting the importance of MACOM-mediated m6A for the regulation of 3′ end processing. Alternatively, MACOM and/or CBLL1 may be regulating CPA indirectly in our experiments via other mechanisms, such as splicing (33,80) or protein ubiquitination (118–120). Demonstrating such potential m6A-dependent or m6A-independent mechanisms for MACOM components to regulate 3′ end processing would require further investigation.

### Involvement of RPRD1B in 3′ end processing

Our screens identified RPRD1B as a weak requirement for CPA and/or termination by RNAP II, in agreement with its affinity for the S2-phosphorylated RNAP II CTD (94), which plays an important role in 3′ end processing (121). The CID of RRPD1B is responsible for this interaction (94,95). This type of interaction is conserved among various RPRD1B homologues, including yeast Rtt103 (122) and the CFII subunit PCF11 (98). Moreover, both RPRD1B and PCF11 use their CIDs to bind RNA at the 3′ ends of genes (4,99,100). This suggests that the CID is a common module for connecting RNAP II with the 3′ ends of mRNA and is used for this purpose by RPRB1B, as well as PCF11.

RNAP II pausing and subsequent release are two steps important for RNAP II recycling and preventing transcriptional readthrough (14,123). Previous studies showed that RPRD1B occupies the 3′ end of the CCND1 gene, and its loss increased RNAP II occupancy distal to the PAS (104). Our study has expanded this phenomenon to a genome-wide scale (Figure 4G, H). These findings are also in agreement with the previous observation that RPRD1B is important for resolving R-loops (92), an RNA-DNA hybrid involved in release of RNAP II at the 3′ends of genes (124). Intriguingly, PCF11 plays a similar role in the dismantling RNAP II transcription complexes (100,125). Therefore, like its homologue PCF11, RPRD1B is a novel factor that controls the release of RNAP II at the 3′ ends of genes. Whether it can do so directly and independently of PCF11 remains to be seen.

### The advantageous attributes of functional high-throughput screens with a dual fluorescence readthrough reporter

In this study, we have developed a dual fluorescence readthrough reporter to assess effects on CPA and carry out screens for human proteins that affect CPA. This system possesses the following advantageous features. First, the dual reporter system effectively minimizes variations in promoter activity or other effects on gene expression that commonly influence single readthrough reporter systems. In this system, the GFP and mCherry reporters are derived from the same mRNA, so that fluctuations in promoter activity among individual cells do not affect the mCherry/GFP ratio. A similar dual fluorescence reporter has been effectively utilized to evaluate protein degradation (48), highlighting the potentially broad usage of such systems. Second, the Flp-In T-REx construct allows for vector integration into a designated genomic locus (47,72,73), avoiding integration site variations. Third, the use of flow cytometry to detect variations in fluorescent reporter expression allows measurement of readthrough effects in individual living cells, enabling the separation of cells with modified 3′ end processing from unaffected ones. Last, this fluorescence-based system is well-suited for genome-scale screens.

Indeed, a combination of this system with pooled genome-wide CRISPR/Cas9 screens resulted in the identification of most of the well-characterized CPA factors. Notably, the significance of gRNA enrichment as measured by padj values in multiple comparisons correlated well with the importance of the known CPA factors. In line with this, gRNAs for most of the well-characterized core CPA complex subunits were enriched with high confidence, except for ones that are already known to have redundant paralogs. Therefore, enrichment significance reflects the known importance of CPA factors for 3′ end processing, providing a semi-quantifiable result. Furthermore, this functional genome-wide screen uncovered both PAS-bound (e.g. CPSF) and unbound CPA factors (e.g. CCNK and CDK12). This is an important advantage over biochemical and proteomic approaches. Thus, this approach can complement biochemical and proteomic methods to identify factors that influence CPA. This technological advancement promises to deepen our comprehension of gene regulation via 3′end processing in various contexts.

## Supplementary Material

gkae240_Supplemental_File

## Data Availability

ICLIP-seq data were deposited to GEO (Gene Expression Omnibus) with accession code GSE230846. gRNA enrichment and RNAP II ChIP-seq data were deposited to GEO with accession code GSE243457.

## References

[1] Colgan D.F. , Manley J.L. Mechanism and regulation of mRNA polyadenylation. Genes Dev. 1997; 11:2755–2766. 9353246 10.1101/gad.11.21.2755

[2] Di Giammartino D.C. , Nishida K., Manley J.L. Mechanisms and consequences of alternative polyadenylation. Mol. Cell. 2011; 43:853–866. 21925375 10.1016/j.molcel.2011.08.017 PMC3194005

[3] Proudfoot N.J. Ending the message: poly(A) signals then and now. Genes Dev. 2011; 25:1770–1782. 21896654 10.1101/gad.17268411 PMC3175714

[4] Xiang K. , Tong L., Manley J.L. Delineating the structural blueprint of the pre-mRNA 3′-end processing machinery. Mol. Cell. Biol. 2014; 34:1894–1910. 24591651 10.1128/MCB.00084-14 PMC4019069

[5] Yang Q. , Doublié S. Structural biology of poly(A) site definition. Wiley Interdiscip. Rev. RNA. 2011; 2:732–747. 21823232 10.1002/wrna.88 PMC3153407

[6] Zhao J. , Hyman L., Moore C. Formation of mRNA 3′ ends in eukaryotes: mechanism, regulation, and interrelationships with other steps in mRNA synthesis. Microbiol. Mol. Biol. Rev. 1999; 63:405–445. 10357856 10.1128/mmbr.63.2.405-445.1999 PMC98971

[7] Tian B. , Manley J.L. Alternative polyadenylation of mRNA precursors. Nat. Rev. Mol. Cell Biol. 2017; 18:18–30. 27677860 10.1038/nrm.2016.116 PMC5483950

[8] Mitschka S. , Mayr C. Context-specific regulation and function of mRNA alternative polyadenylation. Nat. Rev. Mol. Cell Biol. 2022; 23:779. 35798852 10.1038/s41580-022-00507-5 PMC9261900

[9] Danckwardt S. , Hentze M.W., Kulozik A.E. 3′ end mRNA processing: molecular mechanisms and implications for health and disease. EMBO J. 2008; 27:482–498. 18256699 10.1038/sj.emboj.7601932 PMC2241648

[10] Nourse J. , Spada S., Danckwardt S. Emerging roles of RNA 3′-end cleavage and polyadenylation in pathogenesis. Diagn. Ther. Hum. Disord. Biomol. 2020; 10:915. 10.3390/biom10060915 PMC7356254 32560344

[11] Gruber A.J. , Zavolan M. Alternative cleavage and polyadenylation in health and disease. Nat. Rev. Genet. 2019; 20:599–614. 31267064 10.1038/s41576-019-0145-z

[12] Edmonds M. A history of poly A sequences: from formation to factors to function. Prog. Nucleic Acid Res. Mol. Biol. 2002; 71:285–389. 12102557 10.1016/s0079-6603(02)71046-5

[13] Edmonds M. , Vaughan M.H., Nakazato H. Polyadenylic acid sequences in the heterogeneous nuclear RNA and rapidly-labeled polyribosomal RNA of HeLa cells: possible evidence for a precursor relationship. Proc. Natl. Acad. Sci. U.S.A. 1971; 68:1336–1340. 5288383 10.1073/pnas.68.6.1336 PMC389184

[14] Proudfoot N.J. , Furger A., Dye M.J. Integrating mRNA processing with transcription. Cell. 2002; 108:501–512. 11909521 10.1016/s0092-8674(02)00617-7

[15] Proudfoot N.J. Nucleotide sequence from the coding region of rabbit beta-globin messenger RNA. Nucleic Acids Res. 1976; 3:1811–1821. 61580 10.1093/nar/3.7.1811 PMC343038

[16] Shi Y. , Manley J.L. The end of the message: multiple protein-RNA interactions define the mRNA polyadenylation site. Genes Dev. 2015; 29:889–897. 25934501 10.1101/gad.261974.115 PMC4421977

[17] Shi Y. , Di Giammartino D.C., Taylor D., Sarkeshik A., Rice W.J., Yates J.R., Frank J., Manley J.L. Molecular architecture of the human pre-mRNA 3′ processing complex. Mol. Cell. 2009; 33:365–376. 19217410 10.1016/j.molcel.2008.12.028 PMC2946185

[18] Takagaki Y. , Manley J.L. Complex protein interactions within the human polyadenylation machinery identify a novel component. Mol. Cell. Biol. 2000; 20:1515–1525. 10669729 10.1128/mcb.20.5.1515-1525.2000 PMC85326

[19] Ruepp M.D. , Schweingruber C., Kleinschmidt N., Schümperli D Interactions of CstF-64, CstF-77, and symplekin: implications on localisation and function. Mol. Biol. Cell. 2011; 22:91–104. 21119002 10.1091/mbc.E10-06-0543 PMC3016980

[20] Barillà D. , Lee B.A., Proudfoot N.J. Cleavage/polyadenylation factor IA associates with the carboxyl-terminal domain of RNA polymerase II in Saccharomyces cerevisiae. Proc. Natl. Acad. Sci. U.S.A. 2001; 98:445–450. 11149954 10.1073/pnas.98.2.445 PMC14606

[21] Davidson L. , Muniz L., West S. 3′ end formation of pre-mRNA and phosphorylation of Ser2 on the RNA polymerase II CTD are reciprocally coupled in human cells. Genes Dev. 2014; 28:342–356. 24478330 10.1101/gad.231274.113 PMC3937513

[22] Hirose Y. , Manley J.L. RNA polymerase II is an essential mRNA polyadenylation factor. Nature. 1998; 395:93–96. 9738505 10.1038/25786

[23] McCracken S. , Fong N., Yankulov K., Ballantyne S., Pan G., Greenblatt J., Patterson S.D., Wickens M., Bentley D.L. The C-terminal domain of RNA polymerase II couples mRNA processing to transcription. Nature. 1997; 385:357–360. 9002523 10.1038/385357a0

[24] Laishram R.S. , Anderson R.A. The poly A polymerase Star-PAP controls 3′-end cleavage by promoting CPSF interaction and specificity toward the pre-mRNA. EMBO J. 2010; 29:4132–4145. 21102410 10.1038/emboj.2010.287 PMC3018792

[25] Christofori G. , Keller W. Poly(A) polymerase purified from HeLa cell nuclear extract is required for both cleavage and polyadenylation of pre-mRNA in vitro. Mol. Cell. Biol. 1989; 9:193–203. 2538718 10.1128/mcb.9.1.193-203.1989 PMC362161

[26] Scorilas A. Polyadenylate polymerase (PAP) and 3′ end pre-mRNA processing: function, assays, and association with disease. Crit. Rev. Clin. Lab. Sci. 2002; 39:193–224. 12120781 10.1080/10408360290795510

[27] Kühn U. , Gündel M., Knoth A., Kerwitz Y., Rüdel S., Wahle E. Poly(A) tail length is controlled by the nuclear poly(A)-binding protein regulating the interaction between poly(A) polymerase and the cleavage and polyadenylation specificity factor. J. Biol. Chem. 2009; 284:22803–22814. 19509282 10.1074/jbc.M109.018226 PMC2755688

[28] Di Giammartino D.C. , Li W., Ogami K., Yashinskie J.J., Hoque M., Tian B., Manley J.L. RBBP6 isoforms regulate the human polyadenylation machinery and modulate expression of mRNAs with AU-rich 3′ UTRs. Genes Dev. 2014; 28:2248. 25319826 10.1101/gad.245787.114 PMC4201286

[29] Dubbury S.J. , Boutz P.L., Sharp P.A. Cdk12 regulates DNA repair genes by suppressing intronic polyadenylation. Nature. 2018; 564:141. 30487607 10.1038/s41586-018-0758-y PMC6328294

[30] Ciurciu A. , Duncalf L., Jonchere V., Lansdale N., Vasieva O., Glenday P., Rudenko A., Vissi E., Cobbe N., Alphey L. et al. PNUTS/PP1 regulates RNAPII-mediated gene expression and is necessary for developmental growth. PLoS Genet. 2013; 9:e1003885. 24204300 10.1371/journal.pgen.1003885 PMC3814315

[31] Cortazar M.A. , Sheridan R.M., Erickson B., Fong N., Glover-Cutter K., Brannan K., Bentley D.L. Control of RNA pol II speed by PNUTS-PP1 and Spt5 dephosphorylation facilitates termination by a “sitting duck torpedo” mechanism. Mol. Cell. 2019; 76:896. 31677974 10.1016/j.molcel.2019.09.031 PMC6927536

[32] Yue Y. , Liu J., Cui X., Cao J., Luo G., Zhang Z., Cheng T., Gao M., Shu X., Ma H. et al. VIRMA mediates preferential m6A mRNA methylation in 3′UTR and near stop codon and associates with alternative polyadenylation. Cell Discov. 2018; 4:10. 29507755 10.1038/s41421-018-0019-0 PMC5826926

[33] Horiuchi K. , Kawamura T., Iwanari H., Ohashi R., Naito M., Kodama T., Hamakubo T. Identification of Wilms’ tumor 1-associating protein complex and its role in alternative splicing and the cell cycle. J. Biol. Chem. 2013; 288:33292. 24100041 10.1074/jbc.M113.500397 PMC3829175

[34] Knuckles P. , Bühler M. Adenosine methylation as a molecular imprint defining the fate of RNA. FEBS Lett. 2018; 592:2845–2859. 29782652 10.1002/1873-3468.13107 PMC6175371

[35] Albrecht T.R. , Wagner E.J. snRNA 3′ end formation requires heterodimeric association of integrator subunits. Mol. Cell. Biol. 2012; 32:1112–1123. 22252320 10.1128/MCB.06511-11 PMC3295014

[36] Peart N. , Wagner E.J. Gain-of-function reporters for analysis of mRNA 3′-end formation: design and optimization. BioTechniques. 2016; 60:137–140. 26956091 10.2144/000114390

[37] Steinmetz E.J. , Conrad N.K., Brow D.A., Corden J.L. RNA-binding protein Nrd1 directs poly(A)-independent 3′-end formation of RNA polymerase II transcripts. Nature. 2001; 413:327–331. 11565036 10.1038/35095090

[38] Suraweera A. , Lim Y.C., Woods R., Birrell G.W., Nasim T., Becherel O.J., Lavin M.F. Functional role for senataxin, defective in ataxia oculomotor apraxia type 2, in transcriptional regulation. Hum. Mol. Genet. 2009; 18:3384–3396. 19515850 10.1093/hmg/ddp278

[39] Banerjee A. , Sammarco M.C., Ditch S., Wang J., Grabczyk E. A novel tandem reporter quantifies RNA polymerase II termination in mammalian cells. PLoS One. 2009; 4:e6193. 19587781 10.1371/journal.pone.0006193 PMC2702688

[40] Hart T. , Chandrashekhar M., Aregger M., Steinhart Z., Brown K.R., MacLeod G., Mis M., Zimmermann M., Fradet-Turcotte A., Sun S. et al. High-resolution CRISPR screens reveal fitness genes and genotype-specific cancer liabilities. Cell. 2015; 163:1515–1526. 26627737 10.1016/j.cell.2015.11.015

[41] Wang T. , Birsoy K., Hughes N.W., Krupczak K.M., Post Y., Wei J.J., Lander E.S., Sabatini D.M. Identification and characterization of essential genes in the human genome. Science. 2015; 350:1096–1101. 26472758 10.1126/science.aac7041 PMC4662922

[42] Cowley G.S. , Weir B.A., Vazquez F., Tamayo P., Scott J.A., Rusin S., East-Seletsky A., Ali L.D., Gerath W.F.J., Pantel S.E. et al. Parallel genome-scale loss of function screens in 216 cancer cell lines for the identification of context-specific genetic dependencies. Sci. Data. 2014; 1:140035. 25984343 10.1038/sdata.2014.35 PMC4432652

[43] Shalem O. , Sanjana N.E., Hartenian E., Shi X., Scott D.A., Mikkelsen T.S., Heckl D., Ebert B.L., Root D.E., Doench J.G. et al. Genome-scale CRISPR-Cas9 knockout screening in human cells. Science. 2014; 343:84–87. 24336571 10.1126/science.1247005 PMC4089965

[44] Chen S. , Sanjana N.E., Zheng K., Shalem O., Lee K., Shi X., Scott D.A., Song J., Pan J.Q., Weissleder R. et al. Genome-wide CRISPR screen in a mouse model of tumor growth and metastasis. Cell. 2015; 160:1246–1260. 25748654 10.1016/j.cell.2015.02.038 PMC4380877

[45] Lee E.C. , Yu D., Martinez De Velasco J., Tessarollo L., Swing D.A., Court D.L., Jenkins N.A., Copeland N.G. A highly efficient Escherichia coli-based chromosome engineering system adapted for recombinogenic targeting and subcloning of BAC DNA. Genomics. 2001; 73:56–65. 11352566 10.1006/geno.2000.6451

[46] Noordermeer S.M. , Adam S., Setiaputra D., Barazas M., Pettitt S.J., Ling A.K., Olivieri M., Álvarez-Quilón A., Moatti N., Zimmermann M. et al. The shieldin complex mediates 53BP1-dependent DNA repair. Nature. 2018; 560:117–121. 30022168 10.1038/s41586-018-0340-7 PMC6141009

[47] O’Gorman S. , Fox D.T., Wahl G.M. Recombinase-mediated gene activation and site-specific integration in mammalian cells. Science. 1991; 251:1351–1355. 1900642 10.1126/science.1900642

[48] Yen H.C.S. , Xu Q., Chou D.M., Zhao Z., Elledge S.J. Global protein stability profiling in mammalian cells. Science. 2008; 322:918–923. 18988847 10.1126/science.1160489

[49] Ha K.C.H. , Blencowe B.J., Morris Q. QAPA: a new method for the systematic analysis of alternative polyadenylation from RNA-seq data. Genome Biol. 2018; 19:45. 29592814 10.1186/s13059-018-1414-4 PMC5874996

[50] Mak A.B. , Moffat J. A versatile lentiviral expression system to identify mammalian protein-protein interactions. Methods. 2012; 57:409–416. 22713554 10.1016/j.ymeth.2012.06.005

[51] Ni Z. , Olsen J.B., Emili A., Greenblatt J.F. Identification of mammalian protein complexes by lentiviral-based affinity purification and mass spectrometry. Methods Mol. Biol. 2011; 781:31–45. 21877275 10.1007/978-1-61779-276-2_2

[52] Hart T. , Tong A.H.Y., Chan K., Van Leeuwen J., Seetharaman A., Aregger M., Chandrashekhar M., Hustedt N., Seth S., Noonan A. et al. Evaluation and design of genome-wide CRISPR/SpCas9 knockout screens. G3: Genes Genomes Genetics. 2017; 7:2719–2727. 28655737 10.1534/g3.117.041277 PMC5555476

[53] Aregger M. , Chandrashekhar M., Tong A.H.Y., Chan K., Moffat J. Pooled lentiviral CRISPR-Cas9 screens for functional genomics in mammalian cells. Methods Mol. Biol. 2019; 1869:169–188. 30324523 10.1007/978-1-4939-8805-1_15

[54] Huppertz I. , Attig J., D’Ambrogio A., Easton L.E., Sibley C.R., Sugimoto Y., Tajnik M., König J., Ule J. iCLIP: protein-RNA interactions at nucleotide resolution. Methods. 2014; 65:274–287. 24184352 10.1016/j.ymeth.2013.10.011 PMC3988997

[55] Nabeel-Shah S. , Greenblatt J.F. Revised iCLIP-seq protocol for profiling RNA-protein interaction sites at individual nucleotide resolution in living cells. Bio. Protoc. 2023; 13:e4688. 10.21769/BioProtoc.4688 PMC10262072 37323634

[56] Van Nostrand E.L. , Pratt G.A., Shishkin A.A., Gelboin-Burkhart C., Fang M.Y., Sundararaman B., Blue S.M., Nguyen T.B., Surka C., Elkins K. et al. Robust transcriptome-wide discovery of RNA-binding protein binding sites with enhanced CLIP (eCLIP). Nat. Methods. 2016; 13:508–514. 27018577 10.1038/nmeth.3810 PMC4887338

[57] Fillebeen C. , Wilkinson N., Pantopoulos K. Electrophoretic mobility shift assay (EMSA) for the study of RNA-protein interactions: the IRE/IRP example. J. Vis. Exp. 2014; 94:52230. 10.3791/52230 PMC4396942 25548934

[58] Rio D.C. Electrophoretic mobility shift assays for RNA–protein complexes. Cold Spring Harb. Protoc. 2014; 2014:pdb.prot080721. 10.1101/pdb.prot080721 24692495

[59] Schmitges F.W. , Radovani E., Najafabadi H.S., Barazandeh M., Campitelli L.F., Yin Y., Jolma A., Zhong G., Guo H., Kanagalingam T. et al. Multiparameter functional diversity of human C2H2 zinc finger proteins. Genome Res. 2016; 26:1742–1752. 27852650 10.1101/gr.209643.116 PMC5131825

[60] Love M.I. , Huber W., Anders S. Moderated estimation of fold change and dispersion for RNA-seq data with DESeq2. Genome Biol. 2014; 15:550. 25516281 10.1186/s13059-014-0550-8 PMC4302049

[61] Hochberg Y. , Benjamini Y. More powerful procedures for multiple significance testing. Stat. Med. 1990; 9:811–818. 2218183 10.1002/sim.4780090710

[62] Song J. , Nabeel-Shah S., Pu S., Lee H., Braunschweig U., Ni Z., Ahmed N., Marcon E., Zhong G., Ray D. et al. Regulation of alternative polyadenylation by the C2H2-zinc-finger protein Sp1. Mol. Cell. 2022; 82:3135–3150. 35914531 10.1016/j.molcel.2022.06.031

[63] Nabeel-Shah S. , Lee H., Ahmed N., Burke G.L., Farhangmehr S., Ashraf K., Pu S., Braunschweig U., Zhong G., Wei H. et al. SARS-CoV-2 nucleocapsid protein binds host mRNAs and attenuates stress granules to impair host stress response. iScience. 2022; 25:103562. 34901782 10.1016/j.isci.2021.103562 PMC8642831

[64] Bolger A.M. , Lohse M., Usadel B. Trimmomatic: a flexible trimmer for Illumina sequence data. Bioinformatics. 2014; 30:2114. 24695404 10.1093/bioinformatics/btu170 PMC4103590

[65] Trapnell C. , Pachter L., Salzberg S.L. TopHat: discovering splice junctions with RNA-Seq. Bioinformatics. 2009; 25:1105. 19289445 10.1093/bioinformatics/btp120 PMC2672628

[66] Shah A. , Qian Y., Weyn-Vanhentenryck S.M., Zhang C. CLIP Tool Kit (CTK): a flexible and robust pipeline to analyze CLIP sequencing data. Bioinformatics. 2017; 33:566–567. 27797762 10.1093/bioinformatics/btw653 PMC6041811

[67] Feng J. , Liu T., Qin B., Zhang Y., Liu X.S. Identifying ChIP-seq enrichment using MACS. Nat. Protoc. 2012; 7:1728–1740. 22936215 10.1038/nprot.2012.101 PMC3868217

[68] Zhang Y. , Liu T., Meyer C.A., Eeckhoute J., Johnson D.S., Bernstein B.E., Nussbaum C., Myers R.M., Brown M., Li W. et al. Model-based analysis of ChIP-Seq (MACS). Genome Biol. 2008; 9:R137. 18798982 10.1186/gb-2008-9-9-r137 PMC2592715

[69] Pestova T.V. , Kolupaeva V.G., Lomakin I.B., Pilipenko E.V., Shatsky I.N., Agol V.I., Hellen C.U.T. Molecular mechanisms of translation initiation in eukaryotes. Proc. Natl. Acad. Sci. U.S.A. 2001; 98:7029–7036. 11416183 10.1073/pnas.111145798 PMC34618

[70] Ngoi S. , Chien A., Lee C. Exploiting internal ribosome entry sites in gene therapy sector design. Curr. Gene Ther. 2005; 4:15–31. 10.2174/1566523044578095 15032611

[71] Connelly S. , Manley J.L. A functional mRNA polyadenylation signal is required for transcription termination by RNA polymerase II. Genes Dev. 1988; 2:440–452. 2836265 10.1101/gad.2.4.440

[72] Craig N.L. The mechanism of conservative site-specific recombination. Annu. Rev. Genet. 1988; 22:77–105. 3071260 10.1146/annurev.ge.22.120188.000453

[73] Sauer B. Site-specific recombination: developments and applications. Curr. Opin. Biotechnol. 1994; 5:521–527. 7765467 10.1016/0958-1669(94)90068-x

[74] Zhang B. , Liu Y., Liu D., Yang L. Targeting cleavage and polyadenylation specific factor 1 via shRNA inhibits cell proliferation in human ovarian cancer. J. Biosci. 2017; 42:417–425. 29358555 10.1007/s12038-017-9701-x

[75] Mayr C. , Bartel D.P. Widespread shortening of 3′UTRs by alternative cleavage and polyadenylation activates oncogenes in cancer cells. Cell. 2009; 138:673–684. 19703394 10.1016/j.cell.2009.06.016 PMC2819821

[76] Xiang K. , Nagaike T., Xiang S., Kilic T., Beh M.M., Manley J.L., Tong L. Crystal structure of the human symplekin-Ssu72-CTD phosphopeptide complex. Nature. 2010; 467:729–733. 20861839 10.1038/nature09391 PMC3038789

[77] Chan S.L. , Huppertz I., Yao C., Weng L., Moresco J.J., Yates J.R., Ule J., Manley J.L., Shi Y. CPSF30 and Wdr33 directly bind to AAUAAA in mammalian mRNA 3′ processing. Genes Dev. 2014; 28:2370–2380. 25301780 10.1101/gad.250993.114 PMC4215182

[78] Sun Y. , Zhang Y., Hamilton K., Manley J.L., Shi Y., Walz T., Tong L. Molecular basis for the recognition of the human AAUAAA polyadenylation signal. Proc. Natl. Acad. Sci. U.S.A. 2018; 115:E1419–E1428. 29208711 10.1073/pnas.1718723115 PMC5816196

[79] Kamieniarz-Gdula K. , Gdula M.R., Panser K., Nojima T., Monks J., Wiśniewski J.R., Riepsaame J., Brockdorff N., Pauli A., Proudfoot N.J. Selective roles of vertebrate PCF11 in premature and full-length transcript termination. Mol. Cell. 2019; 74:158–172. 30819644 10.1016/j.molcel.2019.01.027 PMC6458999

[80] Horiuchi K. , Kawamura T., Hamakubo T. Wilms’ tumor 1-associating protein complex regulates alternative splicing and polyadenylation at potential G-quadruplex-forming splice site sequences. J. Biol. Chem. 2021; 297:101248. 34582888 10.1016/j.jbc.2021.101248 PMC8605363

[81] Figueroa A. , Fujita Y., Gorospe M. Hacking RNA: hakai promotes tumorigenesis by enhancing the RNA-binding function of PSF. Cell Cycle. 2009; 8:3648–3651. 19855157 10.4161/cc.8.22.9909 PMC2808762

[82] Figueroa A. , Kotani H., Toda Y., Mazan-Mamczarz K., Mueller E.C., Otto A., Disch L., Norman M., Ramdasi R.M., Keshtgar M. et al. Novel roles of hakai in cell proliferation and oncogenesis. Mol. Biol. Cell. 2009; 20:3533–3542. 19535458 10.1091/mbc.E08-08-0845 PMC2719571

[83] Kaneko S. , Rozenblatt-Rosen O., Meyerson M., Manley J.L. The multifunctional protein p54nrb/PSF recruits the exonuclease XRN2 to facilitate pre-mRNA 3′ processing and transcription termination. Genes Dev. 2007; 21:1779–1789. 17639083 10.1101/gad.1565207 PMC1920172

[84] West S. , Gromak N., Proudfoot N.J. Human 5′ → 3′ exonuclease Xrn2 promotes transcription termination at co-transcriptional cleavage sites. Nature. 2004; 432:522–525. 15565158 10.1038/nature03035

[85] Takagaki Y. , Manley J.L. RNA recognition by the human polyadenylation factor CstF. Mol. Cell. Biol. 1997; 17:3907–3914. 9199325 10.1128/mcb.17.7.3907 PMC232243

[86] Yao C. , Choi E.A., Weng L., Xie X., Wan J.I., Xing Y.I., Moresco J.J., Tu P.G., Yates J.R., Shi Y. Overlapping and distinct functions of CstF64 and CstF64τ in mammalian mRNA 3′ processing. RNA. 2013; 19:1781–1790. 24149845 10.1261/rna.042317.113 PMC3884657

[87] Zhu Y. , Wang X., Forouzmand E., Jeong J., Qiao F., Sowd G.A., Engelman A.N., Xie X., Hertel K.J., Shi Y. Molecular mechanisms for CFIm-mediated regulation of mRNA alternative polyadenylation. Mol. Cell. 2018; 69:62–74. 29276085 10.1016/j.molcel.2017.11.031 PMC5756121

[88] Laishram R.S. Poly(A) polymerase (PAP) diversity in gene expression - Star-PAP vs canonical PAP. FEBS Lett. 2014; 588:2185–2197. 24873880 10.1016/j.febslet.2014.05.029 PMC6309179

[89] Eifler T.T. , Shao W., Bartholomeeusen K., Fujinaga K., Jäger S., Johnson J.R., Luo Z., Krogan N.J., Peterlin B.M. Cyclin-dependent kinase 12 increases 3′ end processing of growth factor-induced c-FOS transcripts. Mol. Cell. Biol. 2015; 35:468–478. 25384976 10.1128/MCB.01157-14 PMC4272423

[90] Krajewska M. , Dries R., Grassetti A.V., Dust S., Gao Y., Huang H., Sharma B., Day D.S., Kwiatkowski N., Pomaville M. et al. CDK12 loss in cancer cells affects DNA damage response genes through premature cleavage and polyadenylation. Nat. Commun. 2019; 10:1757. 30988284 10.1038/s41467-019-09703-y PMC6465371

[91] Sun R. , Wei T., Ding D., Zhang J., Chen S., He H.H., Wang L., Huang H. CYCLIN K down-regulation induces androgen receptor gene intronic polyadenylation, variant expression and PARP inhibitor vulnerability in castration-resistant prostate cancer. Proc. Natl. Acad. Sci. U.S.A. 2022; 119:e2205509119. 36129942 10.1073/pnas.2205509119 PMC9522376

[92] Morales J.C. , Richard P., Rommel A., Fattah F.J., Motea E.A., Patidar P.L., Xiao L., Leskov K., Wu S.Y., Hittelman W.N. et al. Kub5-Hera, the human Rtt103 homolog, plays dual functional roles in transcription termination and DNA repair. Nucleic Acids Res. 2014; 42:4996–5006. 24589584 10.1093/nar/gku160 PMC4005673

[93] Stagsted L.V.W. , O’leary E.T., Ebbesen K.K., Hansen T.B. The RNA-binding protein SFPQ preserves long-intron splicing and regulates circRNA biogenesis in mammals. eLife. 2021; 10:e63088. 33476259 10.7554/eLife.63088 PMC7819710

[94] Ni Z. , Xu C., Guo X., Hunter G.O., Kuznetsova O.V., Tempel W., Marcon E., Zhong G., Guo H., Kuo W.H.W. et al. RPRD1A and RPRD1B are human RNA polymerase II C-terminal domain scaffolds for Ser5 dephosphorylation. Nat. Struct. Mol. Biol. 2014; 21:686–695. 24997600 10.1038/nsmb.2853 PMC4124035

[95] Li M. , Ma D., Chang Z. Current understanding of CREPT and p15RS, carboxy-terminal domain (CTD)-interacting proteins, in human cancers. Oncogene. 2021; 40:705–716. 33239754 10.1038/s41388-020-01544-0

[96] Kim M. , Krogan N.J., Vasiljeva L., Rando O.J., Nedea E., Greenblatt J.F., Buratowski S. The yeast Rat1 exonuclease promotes transcription termination by RNA polymerase II. Nature. 2004; 432:517–522. 15565157 10.1038/nature03041

[97] Cui M. , Allen M.A., Larsen A., MacMorris M., Han M., Blumenthal T. Genes involved in pre-mRNA 3′-end formation and transcription termination revealed by a lin-15 operon Muv suppressor screen. Proc. Natl. Acad. Sci. U.S.A. 2008; 105:16665–16670. 18946043 10.1073/pnas.0807104105 PMC2571909

[98] Meinhart A. , Cramer P. Recognition of RNA polymerase II carboxy-terminal domain by 3′-RNA-processing factors. Nature. 2004; 430:223–226. 15241417 10.1038/nature02679

[99] Hollingworth D. , Noble C.G., Taylor I.A., Ramos A. RNA polymerase II CTD phosphopeptides compete with RNA for the interaction with Pcf11. RNA. 2006; 12:555. 16497660 10.1261/rna.2304506 PMC1421100

[100] Zhang Z. , Fu J., Gilmour D.S. CTD-dependent dismantling of the RNA polymerase II elongation complex by the pre-mRNA 3′-end processing factor, Pcf11. Genes Dev. 2005; 19:1572. 15998810 10.1101/gad.1296305 PMC1172063

[101] Enriquez-Harris P. , Levitt N., Briggs D., Proudfoot N.J. A pause site for RNA polymerase II is associated with termination of transcription. EMBO J. 1991; 10:1833–1842. 2050120 10.1002/j.1460-2075.1991.tb07709.x PMC452858

[102] Gromak N. , West S., Proudfoot N.J. Pause sites promote transcriptional termination of mammalian RNA polymerase II. Mol. Cell. Biol. 2006; 26:3986–3996. 16648491 10.1128/MCB.26.10.3986-3996.2006 PMC1488997

[103] Logan J. , Falck-Pedersen E., Darnell J.E., Shenk T. A poly(A) addition site and a downstream termination region are required for efficient cessation of transcription by RNA polymerase II in the mouse beta maj-globin gene. Proc. Natl. Acad. Sci. U.S.A. 1987; 84:8306–8310. 3479794 10.1073/pnas.84.23.8306 PMC299531

[104] Lu D. , Wu Y., Wang Y., Ren F., Wang D., Su F., Zhang Y., Yang X., Jin G., Hao X. et al. CREPT accelerates tumorigenesis by regulating the transcription of cell-cycle-related genes. Cancer Cell. 2012; 21:92–104. 22264791 10.1016/j.ccr.2011.12.016

[105] Bösken C.A. , Farnung L., Hintermair C., Schachter M.M., Vogel-Bachmayr K., Blazek D., Anand K., Fisher R.P., Eick D., Geyer M. The structure and substrate specificity of human Cdk12/Cyclin K. Nat. Commun. 2014; 5:3505. 24662513 10.1038/ncomms4505 PMC3973122

[106] Cheng S.-W.G. , Kuzyk M.A., Moradian A., Ichu T.-A., Chang V.C.-D., Tien J.F., Vollett S.E., Griffith M., Marra M.A., Morin G.B. Interaction of cyclin-dependent kinase 12/CrkRS with cyclin K1 is required for the phosphorylation of the C-terminal domain of RNA polymerase II. Mol. Cell. Biol. 2012; 32:4691–4704. 22988298 10.1128/MCB.06267-11 PMC3486194

[107] Bartkowiak B. , Liu P., Phatnani H.P., Fuda N.J., Cooper J.J., Price D.H., Adelman K., Lis J.T., Greenleaf A.L. CDK12 is a transcription elongation-associated CTD kinase, the metazoan ortholog of yeast Ctk1. Genes Dev. 2010; 24:2303–2316. 20952539 10.1101/gad.1968210 PMC2956209

[108] Greenleaf A.L. Human CDK12 and CDK13, multi-tasking CTD kinases for the new millenium. Transcription. 2019; 10:91–110. 30319007 10.1080/21541264.2018.1535211 PMC6602566

[109] Buratowski S. Connections between mRNA 3′ end processing and transcription termination. Curr. Opin. Cell Biol. 2005; 17:257–261. 15901494 10.1016/j.ceb.2005.04.003

[110] Blazek D. , Kohoutek J., Bartholomeeusen K., Johansen E., Hulinkova P., Luo Z., Cimermancic P., Ule J., Peterlin B.M. The Cyclin K/Cdk12 complex maintains genomic stability via regulation of expression of DNA damage response genes. Genes Dev. 2011; 25:2158–2172. 22012619 10.1101/gad.16962311 PMC3205586

[111] Roignant J.Y. , Soller M. m6A in mRNA: an ancient mechanism for fine-tuning gene expression. Trends Genet. 2017; 33:380–390. 28499622 10.1016/j.tig.2017.04.003

[112] Ke S. , Alemu E.A., Mertens C., Gantman E.C., Fak J.J., Mele A., Haripal B., Zucker-Scharff I., Moore M.J., Park C.Y. et al. A majority of m6A residues are in the last exons, allowing the potential for 3′ UTR regulation. Genes Dev. 2015; 29:2037–2053. 26404942 10.1101/gad.269415.115 PMC4604345

[113] Slobodin B. , Bahat A., Sehrawat U., Becker-Herman S., Zuckerman B., Weiss A.N., Han R., Elkon R., Agami R., Ulitsky I. et al. Transcription dynamics regulate poly(A) tails and expression of the RNA degradation machinery to balance mRNA levels. Mol. Cell. 2020; 78:434–444. 32294471 10.1016/j.molcel.2020.03.022

[114] Shi H. , Wei J., He C. Where, when, and how: context-dependent functions of RNA methylation writers, readers, and erasers. Mol. Cell. 2019; 74:640–650. 31100245 10.1016/j.molcel.2019.04.025 PMC6527355

[115] Roost C. , Lynch S.R., Batista P.J., Qu K., Chang H.Y., Kool E.T. Structure and thermodynamics of N6-methyladenosine in RNA: a spring-loaded base modification. J. Am. Chem. Soc. 2015; 137:2107–2115. 25611135 10.1021/ja513080v PMC4405242

[116] Liu N. , Dai Q., Zheng G., He C., Parisien M., Pan T. N(6)-methyladenosine-dependent RNA structural switches regulate RNA-protein interactions. Nature. 2015; 518:560–564. 25719671 10.1038/nature14234 PMC4355918

[117] Spitale R.C. , Flynn R.A., Zhang Q.C., Crisalli P., Lee B., Jung J.W., Kuchelmeister H.Y., Batista P.J., Torre E.A., Kool E.T. et al. Structural imprints in vivo decode RNA regulatory mechanisms. Nature. 2015; 519:486–490. 25799993 10.1038/nature14263 PMC4376618

[118] Fujita Y. , Krause G., Scheffner M., Zechner D., Leddy H.E.M., Behrens J., Sommer T., Birchmeier W. Hakai, a c-Cbl-like protein, ubiquitinates and induces endocytosis of the E-cadherin complex. Nat. Cell Biol. 2002; 4:222–231. 11836526 10.1038/ncb758

[119] Joazeiro C.A.P. , Wing S.S., Huang H.K., Leverson J.D., Hunter T., Liu Y.C. The tyrosine kinase negative regulator c-Cbl as a RING-type, E2- dependent ubiquitin-protein ligase. Science. 1999; 286:309–312. 10514377 10.1126/science.286.5438.309

[120] Levkowitz G. , Waterman H., Ettenberg S.A., Katz M., Tsygankov A.Y., Alroy I., Lavi S., Iwai K., Reiss Y., Ciechanover A. et al. Ubiquitin ligase activity and tyrosine phosphorylation underlie suppression of growth factor signaling by c-Cbl/Sli-1. Mol. Cell. 1999; 4:1029–1040. 10635327 10.1016/s1097-2765(00)80231-2

[121] Ahn S.H. , Kim M., Buratowski S. Phosphorylation of serine 2 within the RNA polymerase II C-terminal domain couples transcription and 3′ end processing. Mol. Cell. 2004; 13:67–76. 14731395 10.1016/s1097-2765(03)00492-1

[122] Jasnovidova O. , Klumpler T., Kubicek K., Kalynych S., Plevka P., Stefl R. Structure and dynamics of the RNAPII CTDsome with Rtt103. Proc. Natl. Acad. Sci. U.S.A. 2017; 114:11133–11138. 29073019 10.1073/pnas.1712450114 PMC5651779

[123] Park N.J. , Tsao D.C., Martinson H.G. The two steps of poly(A)-dependent termination, pausing and release, can be uncoupled by truncation of the RNA polymerase II carboxyl-terminal repeat domain. Mol. Cell. Biol. 2004; 24:4092–4103. 15121832 10.1128/MCB.24.10.4092-4103.2004 PMC400489

[124] Yanling Zhao D. , Gish G., Braunschweig U., Li Y., Ni Z., Schmitges F.W., Zhong G., Liu K., Li W., Moffat J. et al. SMN and symmetric arginine dimethylation of RNA polymerase II C-terminal domain control termination. Nature. 2016; 529:48–53. 26700805 10.1038/nature16469

[125] Sadowski M. , Dichtl B., Hübner W., Keller W. Independent functions of yeast Pcf11p in pre-mRNA 3′ end processing and in transcription termination. EMBO J. 2003; 22:2167–2177. 12727883 10.1093/emboj/cdg200 PMC156072

[126] Chan S. , Choi E.A., Shi Y. Pre-mRNA 3′-end processing complex assembly and function. Wiley Interdiscip. Rev. RNA. 2011; 2:321–335. 21957020 10.1002/wrna.54 PMC3980678

[127] Vijayakumar A. , Park A., Steitz J.A. Modulation of mRNA 3′-end processing and transcription termination in virus-infected cells. Front. Immunol. 2022; 13:828665. 35222412 10.3389/fimmu.2022.828665 PMC8866245

[128] Lee J.H. , You J., Dobrota E., Skalnik D.G. Identification and characterization of a novel human PP1 phosphatase complex. J. Biol. Chem. 2010; 285:24466–24476. 20516061 10.1074/jbc.M110.109801 PMC2915683

