# Supplementary material for: Identifying human pre-mRNA cleavage and polyadenylation factors by genome-wide CRISPR screens using a dual fluorescence readthrough reporter
Source: Nucleic Acids Res. 2024 Apr 8;52(8):4483–501. doi: 10.1093/nar/gkae240 (PMC11077057; doi:10.1093/nar/gkae240)
Supplement: gkae240_Supplemental_File [file gkae240_supplemental_file.pdf]

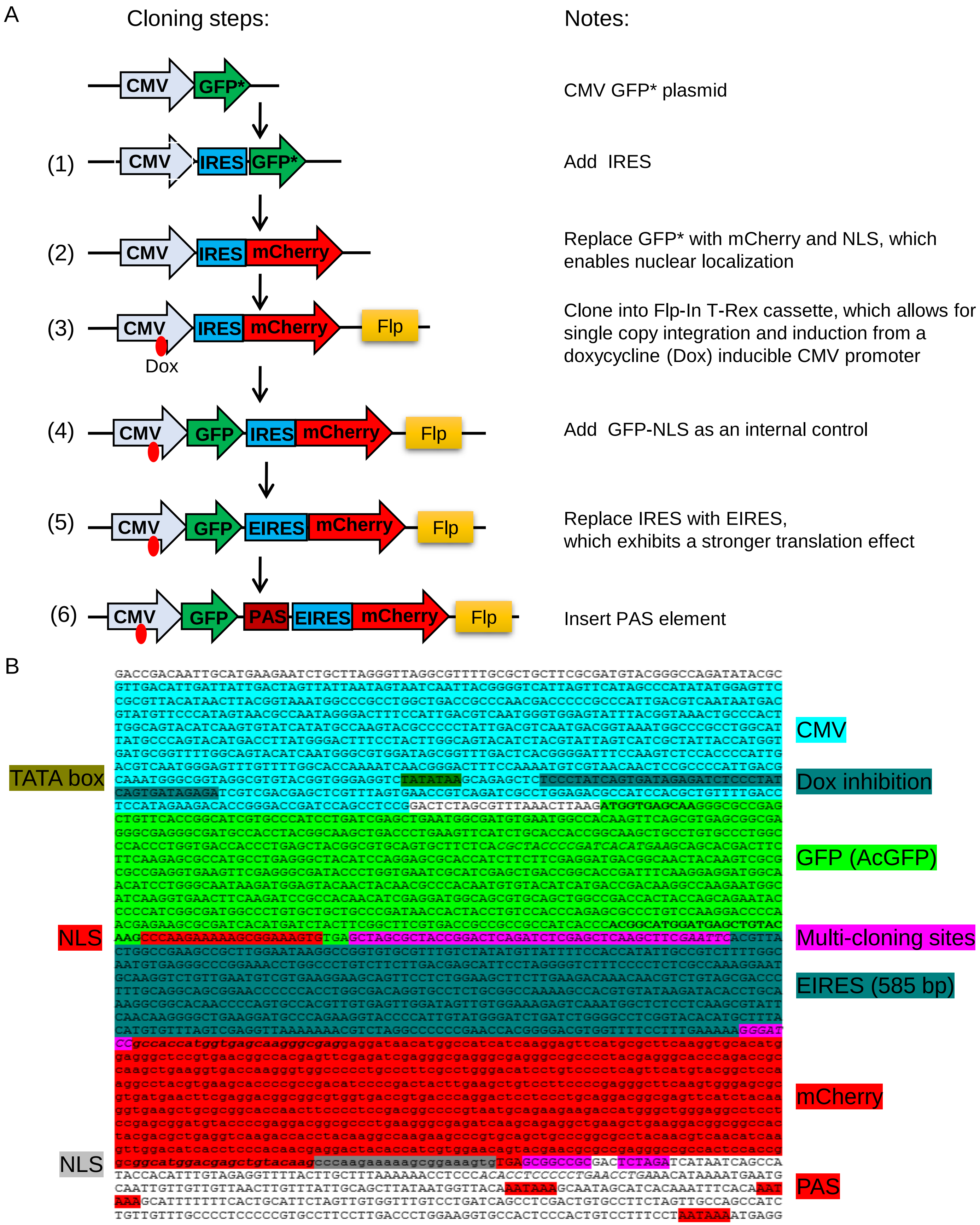

Supplementary Figure S1. Construction of a dual fluorescence readthrough reporter. (**A**) Cloning steps to construct a dual fluorescence readthrough reporter (see Methods for a detailed description). GFP*: GFP without NLS (nuclear localization signal). (**B**) Sequences of the key features cloned into the dual fluorescence readthrough reporter.

A

```
IRES    -----------------------CACGTTACTGGCCGAAGCCGCTTGGAATAAGGCCGGT
EIRES   GCCCCTCTCCCTCCCCCCCCCCTAACGTTACTGGCCGAAGCCGCTTGGAATAAGGCCGGT
                               .************************************

IRES    GTGCGTTTGTCTATATGTTATTTTCCACCATATTGCCGTCTTTTGGCAATGTGAGGGCCC
EIRES   GTGCGTTTGTCTATATGTTATTTTCCACCATATTGCCGTCTTTTGGCAATGTGAGGGCCC
        ************************************************************

IRES    GGAAACCTGGCCCTGTCTTCTTGACGAGCATTCCTAGGGGTCTTTCCCCTCTCGCCAAAG
EIRES   GGAAACCTGGCCCTGTCTTCTTGACGAGCATTCCTAGGGGTCTTTCCCCTCTCGCCAAAG
        ************************************************************

IRES    GAATGCAAGGTCTGTTGAATGTCGTGAAGGAAGCAGTTCCTCTGGAAGCTTCTTGAAGAC
EIRES   GAATGCAAGGTCTGTTGAATGTCGTGAAGGAAGCAGTTCCTCTGGAAGCTTCTTGAAGAC
        ************************************************************

IRES    AAACAACGTCTGTAGCGACCCTTTGCAGGCAGCGGAACCCCCCACCTGGCGACAGGTGCC
EIRES   AAACAACGTCTGTAGCGACCCTTTGCAGGCAGCGGAACCCCCCACCTGGCGACAGGTGCC
        ************************************************************

IRES    TCTGCGGCCAAAAGCCACGTGTATAAGATACACCTGCAAAGGCGGCACAACCCCAGTGCC
EIRES   TCTGCGGCCAAAAGCCACGTGTATAAGATACACCTGCAAAGGCGGCACAACCCCAGTGCC
        ************************************************************

IRES    ACGTTGTGAGTTGGATAGTTGTGGAAAGAGTCAAATGGCTCTCCTCAAGCGTATTCAACA
EIRES   ACGTTGTGAGTTGGATAGTTGTGGAAAGAGTCAAATGGCTCTCCTCAAGCGTATTCAACA
        ************************************************************

IRES    AGGGGCTGAAGGATGCCCAGAAGGTACCCCATTGTATGGGATCTGATCTGGGGCCTCGGT
EIRES   AGGGGCTGAAGGATGCCCAGAAGGTACCCCATTGTATGGGATCTGATCTGGGGCCTCGGT
        ************************************************************

IRES    ACACATGCTTTACATGTGTTTAGTCGAGGTTAAAAAAACGTCTAGGCCCCCCGAACCACG
EIRES   GCACATGCTTTACATGTGTTTAGTCGAGGTTAAAAAAACGTCTAGGCCCCCCGAACCACG
        .***********************************************************

IRES    GGGACGTGGTTTTCCTTTGAAAAA---------------------
EIRES   GGGACGTGGTTTTCCTTTGAAAAACACGATGATAATATGGCCACA
        ************************
```

B

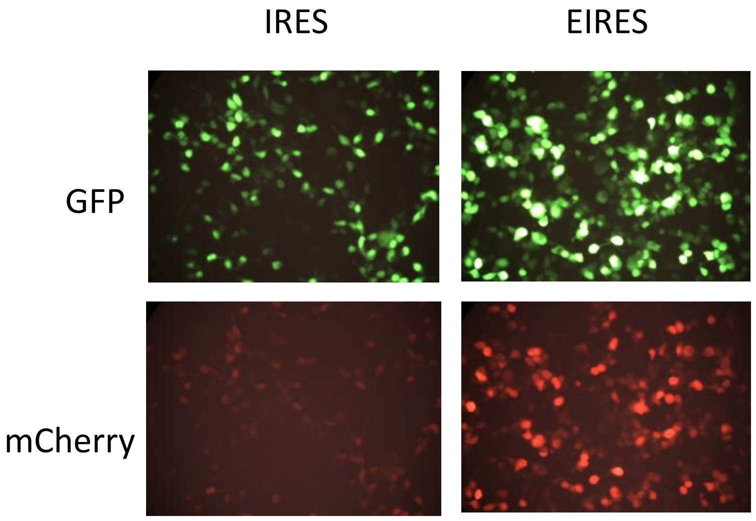

Supplementary Figure S2. Relative translation efficiencies mediated by the IRES and EIRES elements. (**A**) Sequence comparison between the IRES and EIRES elements. Alignment of the sequences between the IRES (540 bp) and EIRES (585 bp) elements that were derivated from the pIGCN21 and MSCV-GPS-GAW plasmids, respectively. (**B**) IRES- and EIRES-mediated expression of mCherry. HEK293 cells were transfected with 500 ng of dual fluorescence readthrough reporter plasmids and induced with 1 µg/ml doxycycline for 24 hours. Images of the expressions of GFP and mCherry were captured by fluorescence microscopy.

A

Hybridized to flowcells | I5 index | Sequencing primers | Vector seq (22 bp) | gRNA

Names	Sequences
D501.F	AATGATACGGCGACCACCGAGATCTACACTATAGCCTACACTCTTTCCCTACACGACGCTCTTCCGATCTTTGTGGAAAGGACGAAACACCG
D502.S.F	AATGATACGGCGACCACCGAGATCTACACATAGAGGCACACTCTTTCCCTACACGACGCTCTTCCGATCTGTTGTGGAAAGGACGAAACACCG
D503.S.F	AATGATACGGCGACCACCGAGATCTACACCCTATCCTACACTCTTTCCCTACACGACGCTCTTCCGATCTCGTTGTGGAAAGGACGAAACACCG
D504.S.F	AATGATACGGCGACCACCGAGATCTACACGGCTCTGAACACTCTTTCCCTACACGACGCTCTTCCGATCTCAGTTGTGGAAAGGACGAAACACCG
D505.S.F	AATGATACGGCGACCACCGAGATCTACACAGGCGAAGACACTCTTTCCCTACACGACGCTCTTCCGATCTGCAGTTGTGGAAAGGACGAAACACCG
D506.S.F	AATGATACGGCGACCACCGAGATCTACACTAATCTTAACACTCTTTCCCTACACGACGCTCTTCCGATCTAGCACTTGTGGAAAGGACGAAACACCG
D507.F	AATGATACGGCGACCACCGAGATCTACACCAGGACGTACACTCTTTCCCTACACGACGCTCTTCCGATCTTTGTGGAAAGGACGAAACACCG
D508.S.F	AATGATACGGCGACCACCGAGATCTACACGTACTGACACACTCTTTCCCTACACGACGCTCTTCCGATCTGTTGTGGAAAGGACGAAACACCG
D509.S.F	AATGATACGGCGACCACCGAGATCTACACTTCGGATGACACTCTTTCCCTACACGACGCTCTTCCGATCTCGTTGTGGAAAGGACGAAACACCG
D510.S.F	AATGATACGGCGACCACCGAGATCTACACACTCATAAACACTCTTTCCCTACACGACGCTCTTCCGATCTCAGTTGTGGAAAGGACGAAACACCG
D511.S.F	AATGATACGGCGACCACCGAGATCTACACGCGCCTCTACACTCTTTCCCTACACGACGCTCTTCCGATCTGCAGTTGTGGAAAGGACGAAACACCG
D512.S.F	AATGATACGGCGACCACCGAGATCTACACCGCGGCTAACACTCTTTCCCTACACGACGCTCTTCCGATCTAGCACTTGTGGAAAGGACGAAACACCG
D513.F	AATGATACGGCGACCACCGAGATCTACACTTATTCGTACACTCTTTCCCTACACGACGCTCTTCCGATCTTTGTGGAAAGGACGAAACACCG
D514.S.F	AATGATACGGCGACCACCGAGATCTACACCCTACGAAACACTCTTTCCCTACACGACGCTCTTCCGATCTGTTGTGGAAAGGACGAAACACCG
D515.S.F	AATGATACGGCGACCACCGAGATCTACACAGCAGATCACACTCTTTCCCTACACGACGCTCTTCCGATCTCGTTGTGGAAAGGACGAAACACCG
D516.S.F	AATGATACGGCGACCACCGAGATCTACACGCGGAGCGACACTCTTTCCCTACACGACGCTCTTCCGATCTCAGTTGTGGAAAGGACGAAACACCG

Staggers

B

Hybridized to flowcells | I7 index | Sequencing primers | Vector seq (22 bp)

Names	Sequences
D701-R	CAAGCAGAAGACGGCATACGAGATCGAGTAATGTGACTGGAGTTCAGACGTGTGCTCTTCCGATCTACTTGCTATTTCTAGCTCTAAAAC
D702-R	CAAGCAGAAGACGGCATACGAGATTCTCCGGAGTGACTGGAGTTCAGACGTGTGCTCTTCCGATCTACTTGCTATTTCTAGCTCTAAAAC
D703-R	CAAGCAGAAGACGGCATACGAGATAATGAGCGGTGACTGGAGTTCAGACGTGTGCTCTTCCGATCTACTTGCTATTTCTAGCTCTAAAAC
D704-R	CAAGCAGAAGACGGCATACGAGATGGAATCTCGTGACTGGAGTTCAGACGTGTGCTCTTCCGATCTACTTGCTATTTCTAGCTCTAAAAC
D705-R	CAAGCAGAAGACGGCATACGAGATTTCTGAATGTGACTGGAGTTCAGACGTGTGCTCTTCCGATCTACTTGCTATTTCTAGCTCTAAAAC
D706-R	CAAGCAGAAGACGGCATACGAGATACGAATTCGTGACTGGAGTTCAGACGTGTGCTCTTCCGATCTACTTGCTATTTCTAGCTCTAAAAC
D707-R	CAAGCAGAAGACGGCATACGAGATAGCTTCAGGTGACTGGAGTTCAGACGTGTGCTCTTCCGATCTACTTGCTATTTCTAGCTCTAAAAC
D708-R	CAAGCAGAAGACGGCATACGAGATGCGCATTAGTGACTGGAGTTCAGACGTGTGCTCTTCCGATCTACTTGCTATTTCTAGCTCTAAAAC
D709-R	CAAGCAGAAGACGGCATACGAGATCATAGCCGGTGACTGGAGTTCAGACGTGTGCTCTTCCGATCTACTTGCTATTTCTAGCTCTAAAAC
D710-R	CAAGCAGAAGACGGCATACGAGATTTCGCGGAGTGACTGGAGTTCAGACGTGTGCTCTTCCGATCTACTTGCTATTTCTAGCTCTAAAAC
D711-R	CAAGCAGAAGACGGCATACGAGATGCGCGAGAGTGACTGGAGTTCAGACGTGTGCTCTTCCGATCTACTTGCTATTTCTAGCTCTAAAAC
D712-R	CAAGCAGAAGACGGCATACGAGATCTATCGCTGTGACTGGAGTTCAGACGTGTGCTCTTCCGATCTACTTGCTATTTCTAGCTCTAAAAC
D713-R	CAAGCAGAAGACGGCATACGAGATGATTATTCGTGACTGGAGTTCAGACGTGTGCTCTTCCGATCTACTTGCTATTTCTAGCTCTAAAAC
D714-R	CAAGCAGAAGACGGCATACGAGATAGCCGCATGTGACTGGAGTTCAGACGTGTGCTCTTCCGATCTACTTGCTATTTCTAGCTCTAAAAC
D715-R	CAAGCAGAAGACGGCATACGAGATCTGATTAAGTGACTGGAGTTCAGACGTGTGCTCTTCCGATCTACTTGCTATTTCTAGCTCTAAAAC
D716-R	CAAGCAGAAGACGGCATACGAGATTAAGCAGTGTGACTGGAGTTCAGACGTGTGCTCTTCCGATCTACTTGCTATTTCTAGCTCTAAAAC

C

i5 index | Upstream annealing | gRNA (20 bp)

AATGATACGGCGACCACCGAGATCTACACTATAGCCTACACTCTTTCCCTACACGACGCTCTTCCGATCTTTGTGGAAAGGACGAAACACCG**NNNNN**
NNNNNNNNNNNNNNNGTTTTAGAGCTAGAAATAGCAAGTAGATCGGAAGAGCACACGTCTGAACTCCAGTCACATTACTCGATCTCGTATGCCGTC
TTCTGCTTG

Downstream annealing | i7 index

Supplementary Figure S3. Illumina barcode sequencing primers and amplicons. (**A**) Illumina TruSeq adapter i5 primers with staggered sequences (~92 bp). (**B**) The primers of the i7 barcode sequences (90 bp). (**C**) The amplicon amplified by the Illumina barcode sequencing primers (~203 bp).

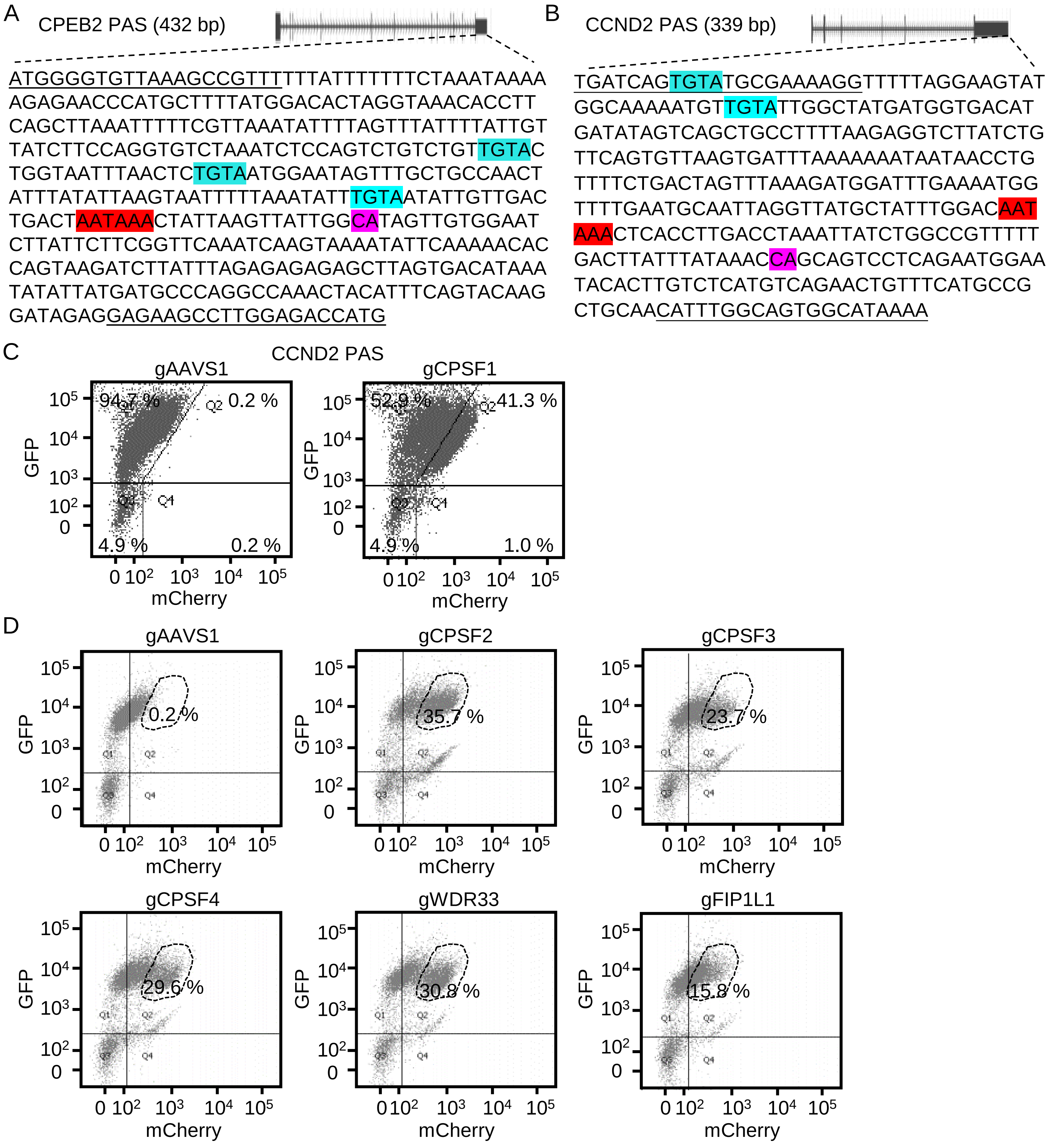

Supplementary Figure S4. PAS elements in the dual fluorescence readthrough reporters increased the mCherry/GFP ratio. (**A** and **B**) PAS DNA elements from the CPEB2 and CCND2 genes, respectively. Red: canonical PAS hexamer sequences. Blue: upstream UGUA sequences. Pink: cleavage sites. Underlined: cloning primer sequences. (**C**) Measurement of readthrough effects in the dual fluorescence readthrough reporter containing the distal PAS of the CCND2 gene. HEK293 cells with the integrated reporter were infected with lentiviruses transducing the indicated gRNAs for 7 days and induced with 1 µg/ml of doxycycline for 4 days. (**D**) Measurement of readthrough effects as carried out in (**C**) in the dual fluorescence readthrough reporter containing the distal PAS of the CPEB2 gene. Circled: mCherry/GFP ratio increased cell population. In (**C**) and (**D**), 10,000 cells were analysed by FACS.

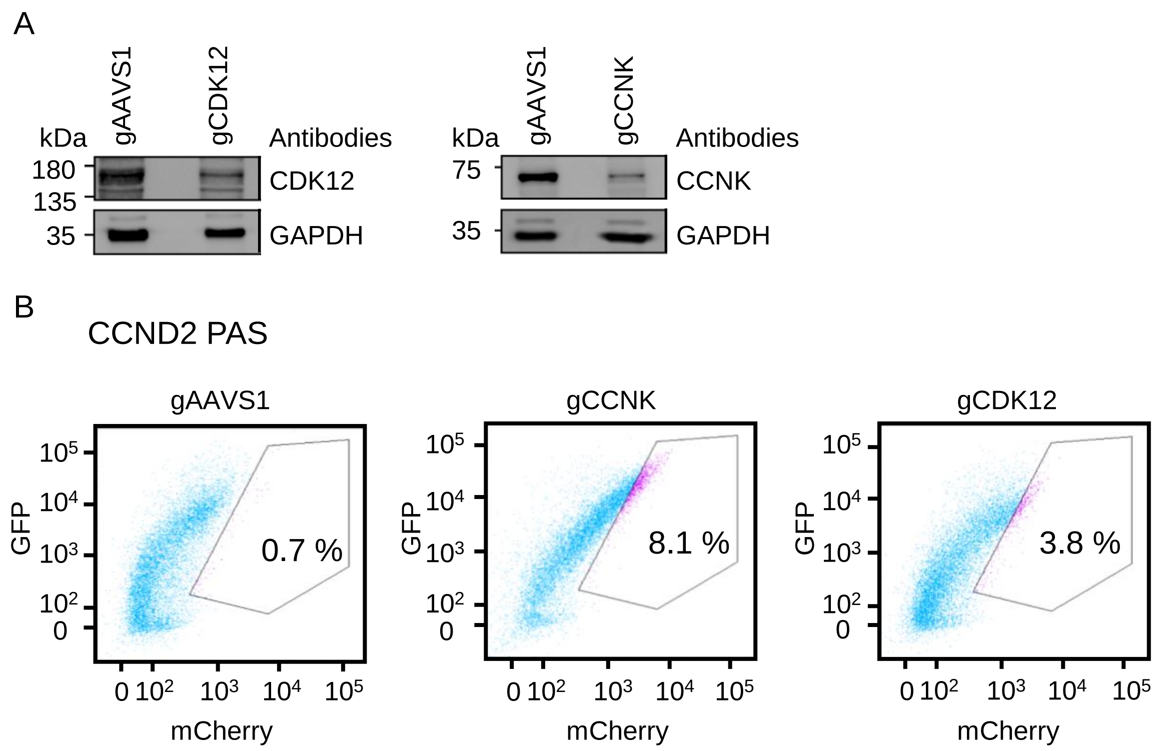

Supplementary Figure S5. Depletions of CDK12 and CCNK increased transcription readthrough effects. (**A**) Depletions of CDK12 and CCNK. Western blots were carried out using the indicated antibodies in lysates from HEK293 cells transduced with indicated lentiviral gRNAs. (**B**) CRISPR/Cas9-mediated depletion of CCNK or CDK12 increases the mCherry/GFP ratio in the dual fluorescence readthrough reporter containing the distal PAS from the CCND2 gene. 10,000 cells were analyzed by FACS. Cell populations with increased mCherry/GFP ratio are shown in pink color within the pentagon.

Supplementary Table 1. The genes identified with the CPEB2 PAS (total of 174 genes)

Genes	baseMean	log2FoldCh	lfcSE	stat	padj
WDR33	5101.608	6.578032	0.239426	27.47414	6.24E-162
CPSF1	5701.31	6.405913	0.292062	21.93343	1.09E-102
CPSF4	6033.873	5.939491	0.274847	21.6102	8.38E-100
CSTF1	2135.183	4.694103	0.502339	9.344489	2.27E-19
CPSF2	1905.717	4.874861	0.648964	7.511762	1.37E-12
SYMPK	1619.537	4.446872	0.68414	6.499944	1.52E-09
CSTF3	1174.03	4.082038	0.66318	6.15525	1.02E-08
NUDT21	1070.123	3.863408	0.661205	5.842982	4.87E-08
FIP1L1	1718.348	4.183923	0.720885	5.803874	5.92E-08
CPSF3	1630.35	4.618001	0.843944	5.471931	3.23E-07
CCNK	726.4752	3.620481	0.834015	4.341025	7.11E-05
CDK12	745.8451	3.520171	0.953812	3.690635	0.000815
PPP1R10	71.77286	2.440611	0.880083	2.773161	0.015508
VIRMA	547.5608	2.433479	0.962565	2.52812	0.029673
PABPN1	384.7281	2.253236	0.936728	2.405434	0.040131
BVES	500.643	1.91673	0.801801	2.39053	0.041608
KRTAP13-3	588.5861	2.541136	1.063511	2.389383	0.041732
HLA-DRA	561.9581	2.496774	1.06345	2.347806	0.046006
SYNGR1	406.3496	2.791863	1.189801	2.346495	0.046155
NKTR	548.5592	2.542559	1.088362	2.336134	0.047302
DPCD	485.2428	2.014689	0.874235	2.304516	0.050809
ANP32A	345.1412	2.447327	1.076897	2.272573	0.05461
FGFBP3	370.1899	2.665863	1.206092	2.210332	0.062631
NAPA	354.92	2.557472	1.164013	2.197117	0.062631
CBLL1	448.739	2.193244	1.005831	2.180529	0.062631
FBXL17	488.952	2.161633	0.998253	2.165416	0.062631
OSGIN1	339.7591	2.369239	1.099804	2.154238	0.062631
RBMS3	387.7365	2.278981	1.06991	2.130069	0.062631
SRD5A3	339.5245	2.356479	1.129479	2.086342	0.062631
NXPH2	452.1843	1.863129	0.896406	2.078442	0.062631
LBP	407.9629	2.078652	1.000926	2.076729	0.062631
GPR146	300.1503	2.218557	1.070898	2.071679	0.062631
EXOSC4	300.6643	2.142375	1.047954	2.044341	0.062631
ZSCAN4	351.5728	2.128478	1.061418	2.005316	0.062631
DTWD2	427.9661	1.885489	0.940372	2.005047	0.062631
PCF11	363.8059	2.100021	1.05265	1.994985	0.062631
HJV	440.9107	2.372762	1.190597	1.992918	0.062631
ITGAE	314.9118	2.379972	1.196884	1.988474	0.062631
RPRD1B	355.5148	2.216194	1.124014	1.971678	0.062631
MBNL3	440.6262	2.095993	1.072552	1.954211	0.062631
FSCN1	392.2545	2.196129	1.126908	1.94881	0.062631
HABP4	390.8338	2.291659	1.189889	1.925943	0.062631
WDR82	429.7226	1.922858	1.002352	1.918347	0.062631
FANCC	293.2937	2.138538	1.117613	1.913486	0.062631

EDN1	331.0605	2.008361	1.05236	1.908436	0.062631
TMEM56	516.1453	2.157732	1.131098	1.907644	0.062631
NCF4	386.0168	2.029298	1.065388	1.904751	0.062631
PPP1R15B	270.8894	2.231783	1.172381	1.903634	0.062631
VIM	347.9705	1.998801	1.050577	1.902574	0.062631
OR14A2	459.6744	1.886042	0.997755	1.890285	0.062631
PRPF4B	376.246	1.889978	1.000799	1.888469	0.062631
IGF2BP2	434.0768	2.253065	1.197093	1.882113	0.062631
BAZ2A	296.256	1.852996	0.985556	1.880153	0.062631
UNC79	437.4266	2.117964	1.129542	1.875065	0.062631
FANCF	378.8241	1.718658	0.926344	1.855313	0.062631
TAFA1	324.648	1.547553	0.844676	1.832126	0.062631
TOX4	421.5582	1.931249	1.055907	1.828996	0.062631
MOB1B	329.0102	2.049502	1.123107	1.824851	0.062631
CDH8	456.6944	2.069802	1.135096	1.82346	0.062631
CHRNA7	355.1142	2.045665	1.122491	1.822433	0.062631
FOXQ1	303.7676	1.741957	0.957686	1.818922	0.062631
SAAL1	308.3925	1.924005	1.05963	1.815734	0.062631
ATOH1	225.7602	1.894884	1.044394	1.814338	0.062631
BOD1L1	366.3743	1.800256	0.994079	1.81098	0.062631
RBPJL	359.6395	1.781765	0.985716	1.807584	0.062631
INKA2	279.8792	1.98822	1.107778	1.794782	0.062631
CCDC174	386.0888	1.662861	0.92784	1.792185	0.062631
PPARG	444.0319	2.008636	1.124071	1.786931	0.062631
RNF145	347.1702	1.798031	1.008485	1.782902	0.062631
HERC1	413.2894	1.86487	1.057152	1.764051	0.062631
SHARPIN	342.3627	1.528685	0.868366	1.760415	0.062631
PPP1R36	322.0054	2.103399	1.195236	1.75982	0.062631
TMEM170B	249.6367	1.720677	0.978602	1.758301	0.062631
HLA-DPB1	416.1282	1.901325	1.082188	1.756927	0.062631
SERPINB2	352.1161	1.881458	1.071034	1.756675	0.062631
PGAP3	313.6815	1.877154	1.069367	1.755388	0.062631
VSNL1	296.2511	1.591824	0.913459	1.742633	0.062631
PYCR3	335.3384	1.707269	0.984362	1.734391	0.062631
OR5B2	354.1187	1.8357	1.062555	1.727628	0.062631
TOP1MT	328.5202	1.806085	1.046998	1.725013	0.062631
HPF1	345.1499	2.039655	1.183565	1.723314	0.062631
TSPO	282.8269	1.556886	0.907012	1.716499	0.062631
AMPD2	394.633	1.682729	0.982426	1.712829	0.062631
DTL	328.2082	1.916246	1.122671	1.706864	0.062631
BNIP3L	376.9582	1.921633	1.126341	1.706084	0.062631
HTRA3	325.5239	1.768434	1.040687	1.699295	0.062631
KRTAP6-2	479.048	1.807618	1.06389	1.699064	0.062631
RCAN2	287.4809	1.881157	1.108201	1.697488	0.062631
TACSTD2	336.8725	1.789701	1.055175	1.696118	0.062631
MFSD2A	304.7119	1.776178	1.048231	1.694453	0.062631
EMC1	317.235	1.559755	0.921262	1.693064	0.062631

EPM2A	423.3813	1.713957	1.015835	1.68724	0.062631
RHPN1	305.8497	1.885348	1.11769	1.686825	0.062631
PHIP	383.1392	1.870306	1.109529	1.685675	0.062631
PALB2	270.901	1.790606	1.065225	1.680966	0.062631
CSF3R	395.2328	1.73425	1.035459	1.674862	0.062631
KCTD21	324.7024	1.86056	1.114447	1.669492	0.062631
CORO1B	411.045	1.89577	1.13636	1.668282	0.062631
PNMA8B	275.2796	1.648298	0.988514	1.66745	0.062631
ZNF169	312.1981	1.958589	1.175449	1.666248	0.062631
CLIC6	236.1492	1.722824	1.035018	1.664535	0.062631
UBXN4	313.4727	1.755147	1.054467	1.664488	0.062631
METTL27	316.472	1.738003	1.050271	1.654814	0.062631
FHL1	364.7983	1.747297	1.05687	1.653275	0.062631
RBM25	305.7719	1.839776	1.117289	1.646642	0.062631
SEMG2	345.1124	1.839396	1.122766	1.638272	0.062631
CLEC3B	284.0622	1.936087	1.182279	1.637589	0.062631
AXIN2	336.4897	1.824724	1.118241	1.631781	0.062631
C17orf77	328.3373	1.729299	1.063735	1.625687	0.062631
TNS4	322.2266	1.707471	1.050345	1.625629	0.062631
CLOCK	266.5421	1.797093	1.105722	1.625266	0.062631
ALDH1B1	333.0464	1.648472	1.017802	1.619639	0.062631
GPN3	297.8909	1.798592	1.112511	1.616696	0.062631
ARX	336.2044	1.805082	1.117151	1.615791	0.062631
CACNA2D1	428.7119	1.841297	1.139598	1.615743	0.062631
KIF23	392.6541	1.91292	1.186201	1.612644	0.062631
AK7	259.7626	1.758159	1.095667	1.604647	0.062631
GFM2	376.4818	1.88998	1.181239	1.599998	0.062631
AGER	332.9367	1.900607	1.190273	1.596782	0.062631
OR52B4	385.402	1.8861	1.182104	1.595545	0.062631
GLE1	175.0869	1.612341	1.020543	1.579885	0.062631
ZC3H3	163.4349	1.60943	1.020097	1.577722	0.062631
HAUS5	246.4935	1.840257	1.171866	1.570365	0.062631
SLX4IP	362.71	1.757377	1.119916	1.569203	0.062631
MFSD3	306.6378	1.710073	1.09195	1.566073	0.062631
EPHX1	323.1791	1.752616	1.119498	1.565537	0.062631
TSKS	292.6927	1.636951	1.046185	1.564685	0.062631
ZNF155	348.5514	1.577032	1.00991	1.561557	0.062631
CPA6	285.7301	1.626205	1.042966	1.559213	0.062631
OR1N2	309.1578	1.63908	1.052164	1.557819	0.062631
YTHDC2	305.0416	1.592671	1.02289	1.557032	0.062631
TECTB	335.3827	1.630267	1.050852	1.551376	0.062631
PTP4A3	368.9406	1.43291	0.92373	1.55122	0.062631
CTHRC1	339.4118	1.627874	1.050962	1.548937	0.062631
MYO1F	420.4413	1.473835	0.952438	1.547435	0.062631
WDR88	285.8206	1.716611	1.109647	1.546988	0.062631
DOCK8	416.6321	1.640681	1.061784	1.545212	0.062631
NOS2	336.7999	1.522994	0.986534	1.543783	0.062631

CD69	354.8963	1.736851	1.125187	1.543611	0.062631
PGK2	387.8707	1.471083	0.956036	1.538732	0.062631
ARRDC1	256.8046	1.604544	1.048219	1.530733	0.062631
BTBD2	239.5978	1.792034	1.1716	1.529561	0.062631
GRK1	273.3689	1.702879	1.11836	1.522658	0.062631
CNN3	296.5257	1.685987	1.108859	1.52047	0.062631
ARPC1B	296.0864	1.784784	1.176861	1.516563	0.062631
NODAL	311.3725	1.49107	0.983232	1.516498	0.062631
TAS2R13	257.109	1.683134	1.110594	1.515526	0.062631
PLEKHH3	281.106	1.581426	1.043668	1.515258	0.062631
ZRANB1	246.0122	1.66697	1.100616	1.514579	0.062631
HMBS	343.6544	1.479857	0.978409	1.512514	0.062631
CD3E	310.8886	1.601822	1.05949	1.511881	0.062631
RPL35	273.5818	1.574773	1.042436	1.510667	0.062631
TXLNA	233.4886	1.474351	0.980506	1.503663	0.062631
CLEC10A	320.591	1.675756	1.114618	1.503435	0.062631
RGL4	310.8749	1.584015	1.056343	1.499527	0.062631
ABCA12	340.5707	1.568768	1.046227	1.499453	0.062631
ENDOD1	239.6129	1.424066	0.952306	1.495388	0.062631
USP6NL	346.2885	1.65398	1.111392	1.488205	0.062631
GATA5	324.2861	1.664708	1.120578	1.48558	0.062631
SLC3A1	366.3297	1.677702	1.130039	1.484641	0.062631
FGFR1OP2	277.4007	1.508025	1.016852	1.483033	0.062631
COLQ	220.8047	1.633975	1.103487	1.480738	0.062631
RNASEH2B	325.0225	1.642727	1.10944	1.480681	0.062631
CYP4F12	309.6315	1.552764	1.050481	1.478146	0.062631
B3GNT7	249.3668	1.491763	1.010367	1.476456	0.062631
LCA5	286.2288	1.729939	1.171902	1.476181	0.062631
CETN2	324.7658	1.731127	1.172901	1.475936	0.062631
NANS	279.58	1.656667	1.123242	1.474897	0.062631
CHAT	246.1622	1.739487	1.181865	1.471816	0.062631
CNPY1	359.5931	1.568611	1.066007	1.471483	0.062631
CXCL10	382.3035	1.646284	1.121832	1.467496	0.062631
OR5A1	327.0448	1.539923	1.050779	1.465505	0.062631
DNAJA3	299.2959	1.610043	1.100246	1.463348	0.062631
VTI1A	283.5253	1.725279	1.179752	1.462408	0.062631

padj < 0.05, 20 genes
padj < 0.25, but > 0.05, 154 genes
Well known core CPA factors, 11 genes
Documented CPA factors, 6 genes
Novel CPA factor charactrized in this study, 1 gene

Supplementary Table 2. The genes identified with the CCND2 PAS (total of 14 genes)

Genes	baseMean	log2FoldChange	lfcSE	stat	padj
WDR33	1531.4	4.592135593	0.252545	18.18344	1.22E-69
CPSF4	1684.397	3.868856136	0.292251	13.23811	4.61E-36
CSTF3	927.9922	3.595344365	0.387647	9.274794	1.04E-16
CPSF1	855.7871	3.456996648	0.388558	8.896994	2.50E-15
CPSF2	973.2974	3.765772566	0.501631	7.507059	1.76E-10
SYMPK	540.3713	2.373666132	0.420357	5.646791	3.57E-05
CSTF1	574.4347	2.623584771	0.473731	5.538135	5.93E-05
CDK12	559.1487	2.965308893	0.554916	5.34371	0.000159
CCNK	603.2625	3.303751383	0.623411	5.299476	0.000161
FIP1L1	958.2688	3.242461926	0.651084	4.9801	0.00076
PCF11	462.9749	2.312962816	0.472223	4.898028	0.001056
NUDT21	362.8481	2.029384517	0.442061	4.590738	0.004281
CPSF3	472.2441	2.597259563	0.611559	4.246947	0.014542
HNRNPU	314.5592	1.676343085	0.484048	3.463177	0.127595

padj < 0.05, 13 genes

padj < 0.25, but > 0.05, 1 gene

Well known core CPA factors, 11 genes

Documented CPA factors, 2 genes
